# *AtTCTP2*, an *Arabidopsis thaliana* homolog of Translationally Controlled Tumor Protein, enhances *in vitro* plant regeneration

**DOI:** 10.3389/fpls.2015.00468

**Published:** 2015-07-02

**Authors:** Roberto Toscano-Morales, Beatriz Xoconostle-Cázares, José L. Cabrera-Ponce, Jesús Hinojosa-Moya, Jorge L. Ruiz-Salas, Santiago V. Galván-Gordillo, Ramón G. Guevara-González, Roberto Ruiz-Medrano

**Affiliations:** ^1^Laboratory of Plant Molecular Biology, Department of Biotechnology and Bioengineering, CINVESTAVMexico City, Mexico; ^2^Department of Plant Genetic Enginneering, CINVESTAV Unidad IrapuatoIrapuato, Mexico; ^3^Facultad de Ingeniería Química, Benemérita Universidad Autónoma de Puebla, Ciudad UniversitariaPuebla, Mexico; ^4^CA Ingenieria de Biosistemas, Centro Universitario Cerro de las Campanas, Universidad Autónoma de QuerétaroSantiago de Querétaro, Mexico

**Keywords:** TCTP, plant regeneration, lateral root, vascular expression, *Agrobacterium rhizogenes*

## Abstract

The Translationally Controlled Tumor Protein (TCTP) is a central regulator of cell proliferation and differentiation in animals, and probably also in plants. Arabidopsis harbors two TCTP genes, *AtTCTP1* (At3g16640), which is an important mitotic regulator, and *AtTCTP2* (At3g05540), which is considered a pseudogene. Nevertheless, we have obtained evidence suggesting that this gene is functional. Indeed, a T-DNA insertion mutant, SALK_045146, displays a lethal phenotype during early rosette stage. Also, both the *AtTCTP2* promoter and structural gene are functional, and heterozygous plants show delayed development. *AtTCTP1* cannot compensate for the loss of *AtTCTP2*, since the accumulation levels of the *AtTCTP1* transcript are even higher in heterozygous plants than in wild-type plants. Leaf explants transformed with *Agrobacterium rhizogenes* harboring *AtTCTP2*, but not *AtTCTP1*, led to whole plant regeneration with a high frequency. Insertion of a sequence present in AtTCTP1 but absent in AtTCTP2 demonstrates that it suppresses the capacity for plant regeneration; also, this phenomenon is enhanced by the presence of TCTP (AtTCTP1 or 2) in the nuclei of root cells. This confirms that *AtTCTP2* is not a pseudogene and suggests the involvement of certain TCTP isoforms in vegetative reproduction in some plant species.

## Introduction

The Translationally Controlled Tumor Protein (TCTP) is a conserved family found in most eukaryotes, which has a central role in growth and developmental regulation. The term derives from the fact that the mRNA is regulated translationally in a tumor cell line, although this does not appear to be general (reviewed in Bommer and Thiele, 2004; Hinojosa-Moya et al., 2008; Nagano-Ito and Ichikawa, 2012; Amson et al., 2013). A wide range of functions has been assigned to these proteins, including regulation of proliferation and programmed cell death, as well as chemokine activities in animals (MacDonald et al., 1995; Kang et al., 2001; Hsu et al., 2007; Susini et al., 2008). The precise role of TCTP is not completely clear, because mutants are pleiotropic; however, its involvement in cell proliferation and development is likely (Amson et al., 2013). The apparent multifunctionality of these proteins is inferred from the fact that they are capable of interacting with diverse targets, including cytoskeletal components, factors involved in cell repair, apoptosis (pro- and antiapoptotic), protein synthesis, and even general metabolism (Amson et al., 2013). In certain cases TCTP displays a non-cell autonomous function in animals, as in the case of the histamine release factor (HRF), which is noteworthy, given the absence of a signal peptide in these proteins (Kang et al., 2001). In fact, TCTP is secreted via a non-classical pathway, for which it requires a peptide transduction domain (Amzallag et al., 2004; Kim et al., 2011). Structurally, TCTP is related to small GTP-binding proteins, and some evidence supports the notion that this protein functions as a guanine nucleotide exchange factor, although through which exact pathway is yet to be determined (Hsu et al., 2007). Silencing of *TCTP* in Drosophila leads to severe developmental defects, most of them related to cell proliferation and cell size. Interestingly, alteration of the putative G-protein binding site leads to inactivation of TCTP (Hsu et al., 2007). There is less information regarding the function of TCTP in plants, but most evidence points to a central role in regulating proliferation and development. Indeed, Arabidopsis *AtTCTP1* (At3g16640) mutants show a lethal phenotype in early stages of embryo development, and silenced lines show defects in cell size, root structure, and pollen (Berkowitz et al., 2008; Brioudes et al., 2010). It is likely that TCTP function in plants is mediated or regulated by auxins (Berkowitz et al., 2008), and that this is probably conserved across kingdoms since Drosophila *TCTP* can rescue an Arabidopsis *TCTP* mutant and vice versa (Brioudes et al., 2010). Interestingly, *AtTCTP1* overexpression also protects against apoptotic cell death triggered by different effectors in plants (Hoepflinger et al., 2013).

As in animals, a plethora of functions is evident for plant TCTPs. For instance, it is required for normal male gametophyte development (Berkowitz et al., 2008), and is involved in response to water deficit, which correlates with its expression in stomata (Kim et al., 2012). It is not known whether TCTP mRNA is also translationally regulated in other systems although the *Pharbitis nil TCTP* mRNA is subject to circadian oscillations (Sage-Ono et al., 1998), and high levels of the *Cucurbita maxima* TCTP (CmTCTP) mRNA, but not protein, are found in the shoot apex (Hinojosa-Moya et al., 2013). Furthermore, TCTP has been found in the phloem exudate proteome of several species (Lin et al., 2009; Rodriguez-Medina et al., 2011), as well as in the phloem transcriptome of lupin (Rodriguez-Medina et al., 2011). Furthermore, we have previously localized the *CmTCTP* mRNA in mature phloem, suggesting a non-cell autonomous function in plants analogous to that in animals, at least in the case of HRF (Hinojosa-Moya et al., 2013).

Most eukaryotes harbor at least one *TCTP* gene; however, multicellular organisms in some cases harbor more than one gene, suggesting a division of labor between different isoforms. Arabidopsis harbors another *TCTP* gene besides *AtTCTP1* termed *AtTCTP2* (At3g05540); since the corresponding mRNA has not been detected and the T-DNA insertion mutant analyzed shows a wild-type phenotype it has been suggested that it is a pseudogene (Berkowitz et al., 2008). However, there is some evidence that this gene is expressed. Preliminary analysis of a T-DNA insertion mutant of this gene indicated severe defects in early postembryonic development. In this work further evidence was found that this gene is indeed functional, and, interestingly, that is capable of inducing whole plant regeneration in tobacco when harbored by *Agrobacterium rhizogenes*, in a manner analogous to *CmTCTP*, and in contrast to *AtTCTP1*. In all, these results indicate that there is a division of labor between TCTP isoforms in Arabidopsis.

## Materials and methods

### Plant material

Arabidopsis ecotype Columbia and *Nicotiana tabacum* (tobacco) ecotype Samsun seeds sown on soil (sterilized soil-peat-agrolite; 2:2:1) were grown in greenhouse conditions under a 16 h light: 8 h dark regime at 25 ± 2°C. Herbicide resistant transgenic plants were screened as follows: seeds were first germinated in soil containing BASTA (1:500; Bayer, http://www.bayer.com/), and then spraying seedlings weekly with BASTA for a period of 4 weeks. For *in vitro* analysis, seeds were sterilized with 70% ethanol for 1 min, 5% sodium hypochlorite for 10 min, and four washes with sterile water. Seeds were sown on MS medium [1.0 MS salts, 1.5% sucrose, and 0.4% agar (Gelrite)] in round plates sealed with microporous tape (3 M) in controlled environment chambers (model PGR15; Conviron, http://www.conviron.com/) under long-day conditions (16 h light/8 h dark). *AtTCTP2* T-DNA insertion line SALK_045146 seeds were obtained from the ABRC collection (https://www.arabidopsis.org/) and grown in parallel on soil in a controlled environment chamber under long-day conditions.

### Molecular biology procedures

To obtain *AtTCTP2* cDNA and genomic ORF, genomic DNA and total RNA were extracted from Arabidopsis Columbia ecotype using the DNAeasy and RNAeasy plant extraction kits (Qiagen, Hilden, Germany) followed by cDNA synthesis, in some cases. These were employed as template using gene specific primer pairs (Table S4) in combination with PCR [Takara ExTaq (Takara USA) with a Biometra T1000 Thermocycler (Biometra AG, Germany)]. Specifically *AtTCTP2* ORF amplification required the use of total RNA extracted from stem and elevated cDNA synthesis temperatures (75°C for 10 min). The 1.5 kb upstream sequence from the start codon of the *AtTCTP2* gene was obtained by PCR from genomic DNA using specific primers (Table S4). The *AtTCTP2* ORF with a stop codon replacing the start codon (*stopAtTCTP2*) was obtained by PCR using a mutagenic primer (Table S4). The *AtTCTP1* and *AtTCTP2* ORFs with the swapped domains (*mAtTCTP1* and *mAtTCTP2*) were obtained from Genscript Corp. (Piscataway, NJ). Fragments selected for specific posttranscriptional gene silencing (PTGS) of *AtTCTP2* and *AtTCTP1* (*AtTCTP2* RNAi and *AtTCTP1* RNAi) were amplified from both 3′ UTR regions (Table S4). All amplicons were then cloned into the pCR8/GW/TOPO vector (Invitrogen) and sequenced to confirm the correct orientation. Then the selected products were recombined into the selected binary vectors [pBGWFS7,0 for promoter analysis (to yield the *ProAtTCTP2:GFP-GUS* construct); pB7FWG2,0 (to yield the *35S:AtTCTP2 ORF-GFP* and *35S:genomic AtTCTP2 ORF-GFP* constructs), pB7FWG,0 (for expression of *ProAtTCTP2:genomic AtTCTP2 ORF*-*GFP*); and pB7GWIWG2(II),0, for gene silencing of *AtTCTP2* and *AtTCTP1*] obtained from Plant Systems Biology (http://www.psb.ugent.be/; University of Ghent, Belgium). The presence of the corresponding insert was analyzed in all cases by digest and PCR. Selected plasmids were finally introduced into *Agrobacterium tumefaciens* (strain C58C1) and/or *Agrobacterium rhizogenes* (strain K599) by electroporation. Positive clones were used for plant transformation.

### Arabidopsis and *N. tabaccum* transformation

Transformation of Arabidopsis plants was carried out using the floral dip method (Clough and Bent, 1998), with some minor modifications. *A. tumefaciens* strain C58C1 harboring the recombinant plasmids were grown on plates containing antibiotic (Spectinomycin 100 mg/L); immature flower buds were then immersed in the bacterial suspension. Seeds were collected and transgenic plants selected for herbicide resistance with BASTA, as previously mentioned. T1 and/or T2 plant lines were used for all analysis.

Transformation of tobacco leaf explants was carried out by co-cultivation and tissue puncture. In both cases cultures containing *A. rhizogenes* strain K599 harboring the recombinant plasmids were grown on selective liquid media (Luria Bertani with Spectinomycin 100 mg/L) during 36 h at 120 rpm; the cultures were concentrated (3000 rpm/1 min) and the resulting pellets re-suspended in the same amount of fresh media. Tobacco leaves 30–45 dag (days after germination) located next to apical or axillary meristems and with a 2–3 cm diameter were selected. Leaves were washed with sterile water and then treated with ethanol 70% (1 min), followed by soaking in 10% sodium hypochlorite for 30 min, and finally washed several times with sterile water. For co-cultivation, a sterile scalpel soaked in the bacterial cultures was used, and for puncture a sterile insulin syringe containing the bacterial culture. After transformation the explants were placed on MS medium [1.0 MS salts, 1.5% sucrose, and 0.4% agar (Gelrite)] and incubated for 3 days in a growth chamber under dark conditions at 25°C, and finally transferred to a controlled environment chamber under long-day conditions (16 h light/8 h dark) during 30 days.

### Complementation of *AtTCTP2* mutant plants

SALK_045146 heterozygous lines were selected by genotyping and grown on soil (30 dag) under controlled growth chamber conditions. These were transformed by the floral dip method (Clough and Bent, 1998) with the *35S::AtTCTP2-GFP* construct harbored by *A. tumefaciens* (C58C1). Seeds were then harvested and selected *in vitro* on MS medium containing 3% sucrose and 25 mM of ammonium glyphosate (Pestanal®; Sigma Aldrich, St. Louis, MO) for 10 days after germination (dag). Selected plants were then transferred to soil and kept in growth chamber (25°C/16 h light-8 h dark) during 10 days. Genomic DNA was extracted (from 20 to 25 mg of tissue per candidate plant) for use as template (100 ng) for detection of the transgene by PCR and also for genotyping (using the primers described in Table S1). Using the LP, RP, and LBb1 primers, a 1062 bp amplification product should be obtained with WT plants, while a 515 bp amplicon should be obtained in homozygous insertional mutants; in the case of heterozygous plants for the T-DNA insertion, both products should be present.

### Quantitative RT-PCR

*AtTCTP2* and *AtTCTP1* RNA levels were determined as follows: total RNA was extracted from a pool of five plants, 50 mg for each tissue (roots, leaves, stems, petioles, apex, and flowers) and used for one-step RT-PCR (10 ng in a 10 μL reaction). A commercial system was used according to the manufacturer's recommendations (KAPA SYBR FAST Universal One-Step qRT-PCR Kit). Specific primers for *AtTCTP2, AtTCTP1*, 18S, and Actin were used (Table S4). The Real Time RT-PCR reactions were incubated in a Rotor Gene 3000 apparatus (Corbett Research, Australia) using the following PCR conditions: 5 min at 42°C for reverse transcription followed by 3 min at 95°C with 45 cycles of denaturation (95°C for 3 s), annealing (58°C for 20 s), and extension (72°C for 3 s). To verify that no additional products were amplified in the reaction, a dissociation curve was generated through progressive sample heating (60–95°C). The Ct value for each product was determined by triplicate in each treatment. 18S rRNA and actin mRNAs were used to normalize gene expression. The comparative ΔΔCT method (Livak and Schmittgen, 2001) was used to determine relative transcript accumulation.

### Measurement of photosynthetic activity

Photosynthetic activity was measured using a LI-6400-XT portable photosynthesis measurement system with a plant Arabidopsis chamber (Li-Cor, Lincoln, NE) in 25-day-old rosette plants growth on sterile soil under 100 μmol m^−2^s^−1^ light intensity at a reference CO_2_ concentration of 600 μmol CO_2_ mol^−1^ with an automatic injector. Photosynthetic activity correction was made relative to the foliar area calculated with the software Image J (rsb.info.nih.gov/ij/).

### Analysis of T-DNA insertion number in SALK_045146 mutants

A quantitative PCR assay was performed with a droplet digital system (QX100, BioRad) to determine of number of T-DNA insertions in the SALK_045146 line, as described in detail previously (Mazaika and Homsy, 2014) and following the manufacturer's recommendations (BioRad). The reaction mixture was as follows: 20 μl of total volume, 250 nM of probe, and 900 nM of each primer (see Table S4), 2× of reaction mixture (ddPCR supermix for probes, BioRad) and 0.5 ng of genomic DNA. The genomic DNA was digested previously with EcoRI. The PCR program was as follows: 95°C during 10 min, after 40 cycles of 94°C during 30 s and 56°C for 1 min, and finally an extension of 10 min at 98°C. Number of insertions was determined by performing the copy number variation (CNV) protocol using the Quantasoft Software (Biorad). Basically, copy number is determined for an endogenous control, in this case HMGB1 (At3g51880), for which there is a single copy in the Arabidopsis genome, and number of copies of the T-DNA (CaMV 35S promoter) in heterozygous SALK_045146 and WT lines (as control), and these lectures are then compared. For sequences of primers and probes see Table S4.

### Histochemical analysis

Three, four, and six week-old plants expressing the *ProAtTCTP2*:*GFP-GUS* construct were used for histochemical studies. Different tissues were selected: roots, source rosette and cauline leaves, stem, and inflorescence were sectioned and incubated overnight, at 37°C, with GUS buffer, as described (Weigel and Glazebrook, 2002). Tissues were examined with a stereomicroscope (National Instruments) and images collected on a Motic camera (Motic, http://www.motic.com/ Motic, Vancouver, Canada). The GUS reaction was considered negative when no stain was detected after a 24 h incubation period.

### Confocal analysis

Transgenic plants expressing the GFP fusion constructs were analyzed with a Leica confocal laser-scanning microscope (model TC-SP5/MO-TANDEM) using a krypton/argon laser and the following filter settings: 488 nm excitation and 525 nm emission for green fluorescence, and 580/665 nm for chlorophyll autofluorescence. All images were recorded and analyzed with Leica Las AF software, followed by processing with Photoshop 8.0 software (Adobe) as described (Xoconostle-Cázares et al., 1999).

### Dry weight kinetics of tobacco calli

Suspension cultures of NT1 tobacco cells were grown and maintained on liquid NT medium comprised of MS salts (Murashige and Skoog, 1962) with slight modifications (Russell et al., 1992). Cultures were kept on an orbital shaker at 26°C in the dark and subcultured at 7-day intervals. The volume of settled cells (SCV) was measured for 10 ml of 7 day-old cells, which were settled for 30 min. Cell number and viability were assessed with trypan blue. Samples with the same SCV were resuspended in 50 ml NT medium for 4 days. About 5 ml of 4 day-old subcultured tobacco cells was collected on filter paper (Whatman No. 1, 5.5 cm) by vacuum filtration. Twenty-four hours before bombardment filters were placed inside 90-mm Petri dishes (Phoenix Biomedical) containing NT1 medium supplied with 0.2 M mannitol and 0.2 M sorbitol to increase transformation efficiency. Plasmids containing the overexpression constructs were purified using a commercial system (Plasmid Midi kit, Qiagen) previous to bombardment. The cells were then bombarded with tungsten particles coated with the different constructs. Microprojectile preparation was performed according to Tomes et al. (1995), modified by Cabrera-Ponce et al. (1997). Cells were bombarded using a Biolistic PDS-1000/Helium Vacuum System (Bio-Rad) according to the manufacturer's instructions at 7 cm from the end of the barrel of the particle gun. Twenty-four hours after bombardment the filters were transferred to NT medium; 15 days later selection was started on NT medium supplemented with 0, 100, 200, and 300 mg/l kanamycin. Bombarded cells were incubated for 15 days at 26°C without light. Finally transformants were maintained on solid NT medium containing kanamycin at 100 mg/l. Dry weight of three independent calli per treatment at each point was determined every 7 days during 3 weeks.

### Effect of different compounds on *AtTCTP1* and *AtTCTP2* mRNA accumulation

This was done essentially as described (Nemhauser et al., 2006). WT Arabidopsis plants were grown for 15 days on solid MS medium (1×; 2% sucrose) in a growth chamber under controlled conditions (25°C; 50% humidity; 100–120 μmol/m^2^s light) until 4–6 true leaves emerged. Later, three plants per treatment were collected and immersed in liquid MS medium (under controlled conditions) during 3 h containing the following concentrations of growth regulators, salts or substrates: (a) kinetin 20 μM (cytokinin), indoleacetic acid (IAA) 10 and 20 μM (auxin), gibberellic acid (GA), 1 and 5 μM (gibberellins), abscisic acid (ABA) 1 and 10 μM, brassinolide (BRAS) 1 and 100 μM (brassinosteroids); (b) mannitol 100 and 300 mM, polyethyleneglycole (PEG) 12% v/v, NaCl 100 and 300 mM, KCl 50 and 150 mM, KNO_3_ 1 mM, NaH_2_PO_4_ 1 μM, and 1 mM (both in MS 0.1X), and cold (4°C incubation). After incubation, plants were removed and immediately frozen with liquid nitrogen for total RNA extraction. The latter were used as templates for real time qRT PCR (as described previously) for *AtTCTP1* and *AtTCTP2* transcripts using 18S rRNA for normalization. Fold-change between different conditions tested relative to wild type control was calculated using the ΔΔCT method (Livak and Schmittgen, 2001). Two biological replicates were performed per condition tested and three technical replicates were also carried out in each case.

## Results

### *AtTCTP2* is a functional gene

**Figure 1:**
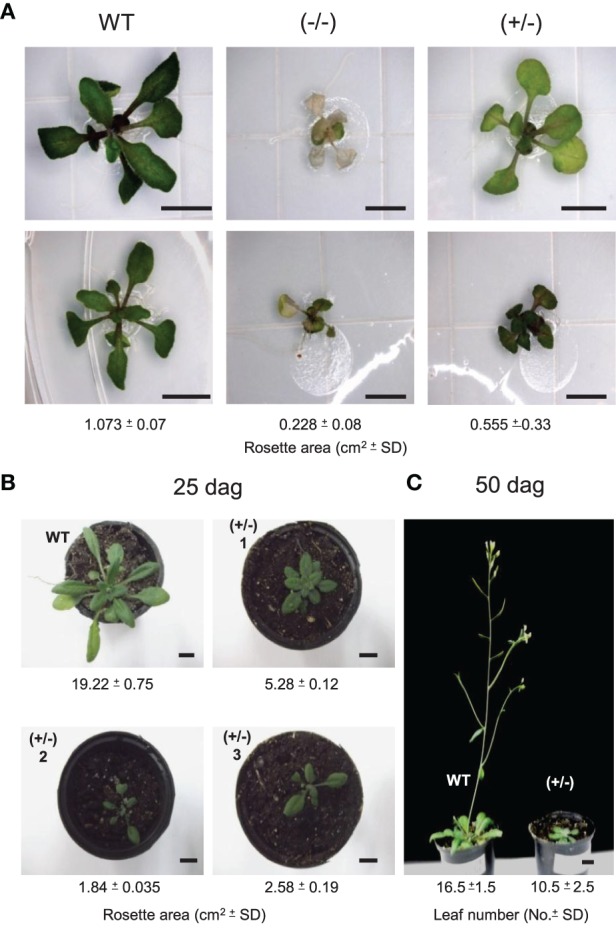
*****AtTCTP2*** insertional mutants (SALK_045146) show severe growth defects in homo- and heterozygosis. (A)** Representative phenotypes of WT, homozygous (-/-) and heterozygous (±) plants grown *in vitro* under standard conditions (10 days after germination). **(B)** Phenotypes of (±) and WT plants grown in soil under standard conditions (see experimental procedures); images were taken 25 days after germination. **(C)** Representative (±) and WT plants 50 days after germination in soil. In all cases, rosette areas and number of leaves were calculated using 5–10 previously genotyped plants; average and SD are shown. Size bars = **(A)** 0.5 cm, **(B)** 1 cm.

**Figure 2:**
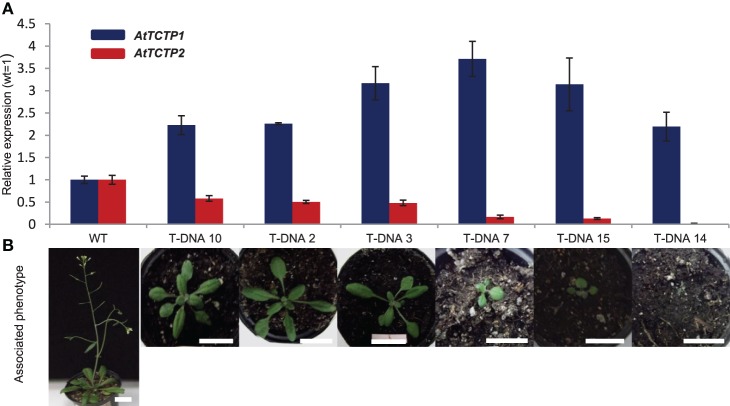
**Phenotypes of heterozygous (±) insertional mutants (SALK_045146) correlate with ***AtTCTP2*** expression levels. (A)** qRT-PCR was performed to determine transcript levels of *AtTCTP2, AtTCTP1*, and 18S rRNA (as endogenous control) using total RNA extracted from several *AtTCTP2* mutants and comparing their expression against WT lines. **(B)** Severity of phenotype of heterozygous (±) *AtTCTP2* mutants ordered by area/size correlates with AtTCTP2 expression levels, and are not associated to a decrease in *AtTCTP1* transcript levels. Both qRT-PCR and analysis of phenotype were performed in plants 25 days after germination. Size bars = 2 cm.

In addition to *AtTCTP1* (At3g16640), Arabidopsis harbors another *TCTP* gene, *AtTCTP2* (At3g05540). RT-PCR assays failed to detect this mRNA, and a T-DNA insertion mutant shows a wild-type phenotype, reasons for which *AtTCTP2* had been considered a pseudogene (Berkowitz et al., 2008). However, another insertional mutant, SALK_045146 (Alonso et al., 2003) shows a lethal phenotype during early stages of development (Figure 1A). Thus, a more thorough analysis was carried out in order to determine whether *AtTCTP2* is a pseudogene. This insertion is located in the fourth exon of the gene; other T-DNA insertions of this gene fall within introns, which could explain their non-lethal phenotype. Two weeks after germination, homozygous SALK_045146 mutants (−/−) developed necrosis in leaves and eventually died (Figure 1A). Other defects were smaller size and fewer rosette leaves. No mutant survived past the early rosette stage, and no intact RNA could be recovered at this stage. Interestingly, heterozygous plants (+/−) displayed an intermediate phenotype. Plants showed a delay in bolting, were shorter and had fewer rosette leaves (Figures 1B,C). Only 15–20% of the heterozygous plants bolted, but only after more than 3 months. This mRNA was quantified to test whether *AtTCTP1* was silenced in (+/−) plants and thus causing the observed phenotype. This could only be carried out in (+/−) plants, because the RNA of (−/−) mutants was quite degraded when the phenotype was evident and thus could not be isolated for RT-PCR. *AtTCTP1* mRNA accumulated to even higher levels than in wild type plants; as expected, *AtTCTP2* levels were lower than in wild type (Figure 2). Moreover, the severity of the phenotype correlated with decreased levels of *AtTCTP2* mRNA in different heterozygous lines, while those for *AtTCTP1* mRNA did not show such correlation, although in all cases were higher than in WT plants (Figure 2). Therefore, loss of one copy of the *AtTCTP2* gene leads to a significant decrease in *AtTCTP2* mRNA, and to a considerable increase in the *AtTCTP1* mRNA. A digital droplet PCR assay was carried out to determine the number of T-DNA insertions in two SALK_045146 heterozygous mutant lines; in these cases only one insertion was found (Figure S1A). Photosynthesis rate, on the other hand, was not affected in heterozygous plants (Figure S1B).

*AtTCTP2* mRNA is present at much lower levels than *AtTCTP1*, according to the AtGenexpress database (http://jsp.weigelworld.org/expviz/expviz.jsp) (3–4 orders of magnitude). Prolonged thermal denaturation, as well as RT-PCR including a small portion of both 5′ and 3′ UTR, followed by a nested PCR using specific *AtTCTP2* ORF primers (Table S4), was required to synthesize *AtTCTP2* cDNA. This suggests that this transcript is rich in secondary structure. The cloned sequence was found to be identical to At3g05540. This thermal denaturation was used to determine the levels of *AtTCTP2* mRNA; indeed, these were ~60-fold in leaves and roots relative to untreated controls, while *AtTCTP1* mRNA was only ~three-fold relative to untreated controls (Figure S2). *AtTCTP2* mRNA accumulated to highest levels in stems, cauline leaves, and roots, while *AtTCTP1* mRNA was more homogeneously distributed between different organs (Figure S3).

### Transformation with *AtTCTP2* complements a knockout mutant

**Figure 3:**
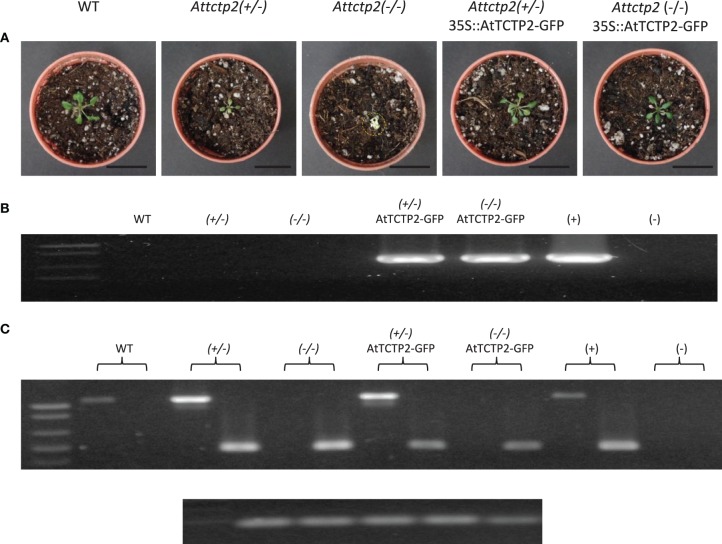
**Transformation with ***AtTCTP2-GFP*** rescues the severe phenotype of heterozygous and homozygous ***AtTCTP2-***knockout mutants. (A)** Phenotype of WT, heterozygous *AtTCTP2*-knockout line [*attctp2* (±)], homozygous *AtTCTP2*-knockout line [*attctp2* (-/-)], *attctp2* (±) knockout line complemented with *35S::AtTCTP2-GFP*, and *attctp2* (-/-) knockout line complemented with *35S::AtTCTP2-GFP*. All plants were selected and grown *in vitro* for 10 days, following soil transfer and acclimation for 10 days, for a total of 20 days after germination (dag). **(B)** Transgene detection by amplification of *GFP* in the same plants. **(C)** Result of genotype analysis of the plants shown in **(A)**. For genotyping, a PCR was carried out with the LP and RP primers, which target the *AtTCTP2* gene and yield a 1062 bp product, and a primer specific for the left border of the T-DNA; the product of the LP and LBb1 primers produce a 515 bp amplicon. Thus, WT plants yield a 1062 bp amplicon corresponding to the *AtTCTP2* gene, homozygous T-DNA insertion mutants for this gene yield a 515 bp product, and heterozygous plants yield both amplicons. 18S rDNA was used as control for DNA integrity (bottom).

To further confirm that the phenotype of the SALK_045146 mutants (homo- and heterozygous) was caused by inactivation of the *AtTCTP2* gene, heterozygous mutants were transformed with the *35S:AtTCTP2-GFP* construct by the floral dip method. Seeds were collected and selected in BASTA, since the original mutant harbors only resistance to kanamycin. Segregants were expected to be WT, heterozygous, and homozygous. Presence of the transgene (*GFP*) was determined by PCR in the segregants; plants that harbored the transgene were also genotyped. It was found that out of 26 seedlings harboring the *35S:AtTCTP2-GFP* construct, 10 were WT, 15 were heterozygous, and one was homozygous. The phenotype of the heterozygous and homozygous plants was similar to WT, indicating that *AtTCTP2* was indeed able to complement these mutants (Figure 3). It is not clear the reason for the deviation from the expected 1:2:1 ratio of WT, heterozygous and homozygous plants, but it must be considered that the 35S promoter is not highly active in ovules, for example (see below, and discussion; Skinner et al., 2004).

### Silencing of *AtTCTP2* results in developmental defects

**Figure 4:**
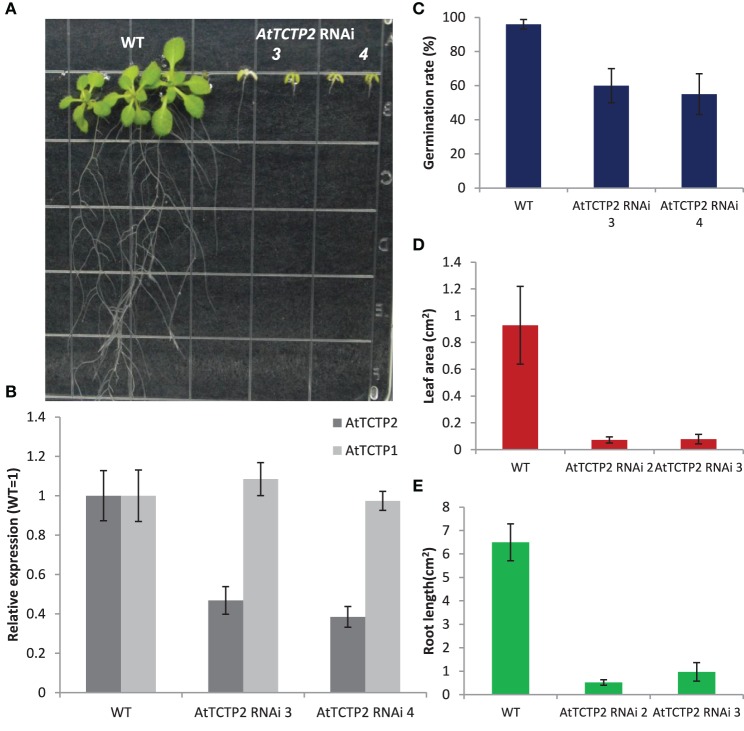
**Silencing of ***AtTCTP2*** results in abnormal phenotypes similar to heterozygous SALK_045146 plants. (A)** Representative phenotypes of *AtTCTP2*-RNAi Arabidopsis lines vs. wild type plants 25 days after germination (dag) on solid MS medium. **(B)** Quantification of *AtTCTP2* and *AtTCTP1* transcripts in both WT and *AtTCTP2*-RNAi lines; qRT-PCRs were performed using as template 50 ng of total RNA extracted from a pool of five randomly selected plants per line; results are shown as *AtTCTP2* transcript levels in *AtTCTP2*-RNAi lines relative to WT (both normalized relative to 18S) using the relative ΔΔCT method described previously. **(C)** Germination rate of both *AtTCTP2*-RNAi lines and WT controls measured 7 dag. **(D)** Leaf area of *AtTCTP2*-RNAi lines and WT controls determined 25 dag. **(E)** Root length of WT controls and both RNAi lines 25 dag. **(C–E)** 30–40 representative plants were selected for analysis; **(D,E)** both measurements were calculated using imageJ free software (http://imagej.nih.gov/ij/). Size bars = 1.1 cm.

To determine the effects of the inactivation of the *AtTCTP2* gene independently from the inactivation of the *AtTCTP1* gene, both were silenced separately by targeting their 3′ untranslated regions. A 200 bp region downstream of the stop codon of both genes was used. Plants harboring the *AtTCTP2* silencing construct were much shorter than WT controls (Figures 4A,C–E), displaying a phenotype similar to heterozygous SALK_045146 mutant lines with more severe defects. The *AtTCTP2* transcript levels in the analyzed lines were roughly 50% that of the WT (Figure 4B). Interestingly, silencing of *AtTCTP1* led to similar phenotypes, although in this case no lateral roots were observed (Figures S4A,B,D–F). The fact that in *AtTCTP2* silenced plants the *AtTCTP1* transcript accumulates to WT or higher levels, and vice versa, suggests that the genes display partially overlapping functions, since the inactivation of one cannot be compensated by the overexpression of the other gene (Figure S4C).

### The *AtTCTP2* upstream region shows a distinct expression pattern different from *AtTCTP1*

**Figure 5:**
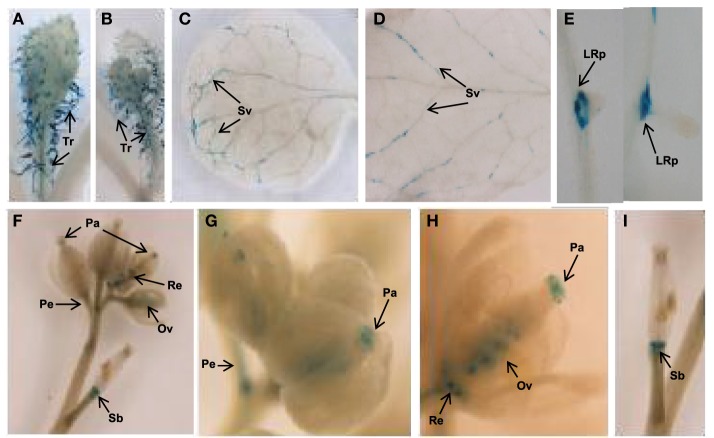
*****AtTCTP2*** promoter region shows a distinctive expression pattern**. Histochemical analysis of Arabidopsis tissues expressing the 1.5 kbp upstream region of the *AtTCTP2* gene directing the expression of GUS-GFP. **(A,B)** GUS activity was detected in trichomes (Tr) of rosette and cauline leaves; **(C,D)** in secondary veins (Sv); **(E)** but also in main and lateral root primordia (LRp) during lateral root formation. **(F)** Signal was also found in closed and open flowers, **(G,H)** particularly in papillae (Pa), ovules (Ov), receptacules (Re), peduncules (Pe), and in **(I)** the base of siliques (Sb), but not in petals.

**Figure 6:**
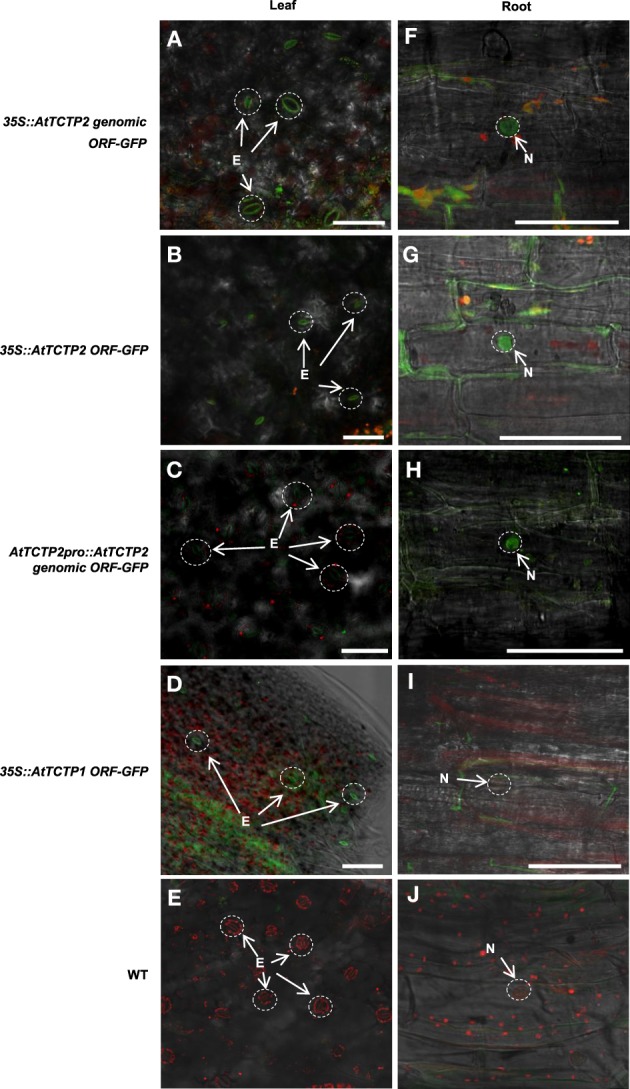
**AtTCTP2-GFP accumulation indicates a correct processing of the ***AtTCTP2*** transcript. (A,F)** *35S::AtTCTP2 genomic* ORF-GFP, **(B,G)** *35S::AtTCTP2 ORF-GFP*, **(C,H)** *AtTCTP2Pro:AtTCTP2-GFP*, and **(D,I)** *35S::AtTCTP1 ORF-GFP* plants. GFP signal was observed in stomata and in root cortex cell nuclei. **(E,J)** WT control showing background fluorescence in leaf and root tissue. Bars = 50 μM.

Next, the expression pattern of the putative *AtTCTP2* gene promoter was analyzed. A 1.5 kb region upstream of the start codon was fused to a *GUS (uidA):GFP* translational fusion in the pFWG2,0 vector. Histochemical analysis indicated that the *AtTCTP2* promoter region is active in segments of minor veins in rosette leaves, vascular tissue of petioles and inflorescence, stems, trichomes, base of siliques, papillae, ovules, and lateral roots (Figures 5A–I). The genomic and processed open reading frame (ORF) fused to GFP, the expression of which was directed by the CaMV 35S promoter, yielded fluorescence in trichome (not shown), stomata, and in mesophyll and root cortex nuclei (Figures 6A,B,F,G). No fluorescence was detected on the control WT plant (Figures 6E,J). Thus, *AtTCTP2* transcripts are probably processed correctly. The accumulation pattern of the AtTCTP2 genomic ORF-GFP fusion was similar when either the CaMV 35S or the endogenous promoter were used (Figures 6C,H), suggesting post-transcriptional regulation of this gene. AtTCTP1:GFP does not localize to root nuclei, indicating functional specialization between both Arabidopsis TCTP isoforms (Figures 6D,I).

### *AtTCTP2* has the capacity to increase biomass and induce whole-plant regeneration when harbored by *A. rhizogenes*

The capacity of *AtTCTP2* to induce an increase in biomass was determined in transformed tobacco calli. Its overexpression induced a moderate albeit reproducible increase (~20%) in biomass relative to empty vector control, as reported for *AtTCTP1*, suggesting that both may regulate proliferation (Figure S5). However, since *AtTCTP1* cannot compensate for the loss of *AtTCTP2*, it is possible that these genes have partially non-overlapping functions. We have shown that the *Cucurbita maxima* phloem *TCTP* (*CmTCTP*) also induces an increase in biomass; additionally, it induces whole plant regeneration in tobacco when harbored by *Agrobacterium rhizogenes* K599 (Hinojosa-Moya et al., 2013). We used this system to determine whether *AtTCTP2* and *AtTCTP1* show regenerating capacity.

**Figure 7:**
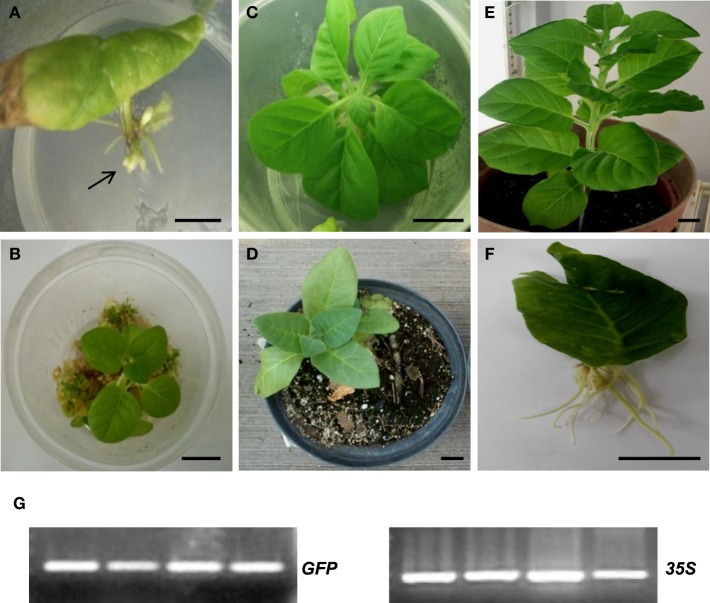
**Inoculation of ***A. rhizogenes*** harboring ***AtTCTP2*** induces plant regeneration in tobacco explants**. Representative images of tobacco regeneration at **(A)** 15, **(B)** 30, and **(C)** 45 days after explant transformation with *AtTCTP2* harbored in *A. rhizogenes*. Examples of regenerated plants transferred to soil at **(D)** 50 and **(E)** 90 days after explant transformation. **(F)** *A. rhizogenes* harboring *AtTCTP1* showed root induction but no plant regeneration. Size bars = 2 cm. **(G)** PCR amplification of *GFP* and *35S* transgenes in representative regenerated plants.

**Figure 8:**
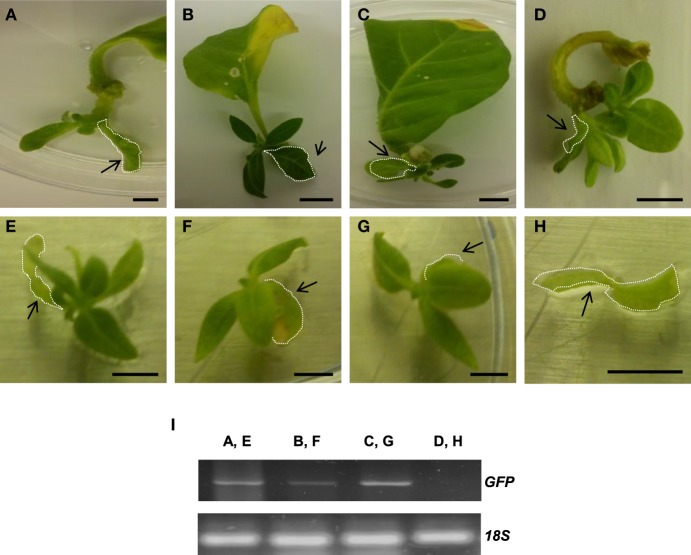
**Plant regeneration occurs in leaves from regenerated tobacco plants harboring ***AtTCTP2***. (A–D)** Representative images of regeneration from four leaf explants 21 days after transformation with the *AtTCTP2*-overexpressing construct harbored by *A. rhizogenes*. Selected leaf explants (dashed) were cut and transferred to fresh solid MS basal medium (without hormone supplementation) and incubated under controlled conditions (16:8 photoperiod). **(E–G)** New tissue arose 21 days after incubation in three samples, while in one of four samples tested **(H)** no self-regeneration capacity was observed. Original explants are highlighted in arrowheads. **(I)** GFP amplification by final point PCR using total DNA extracted from each original explant (arrowheads) as template to test transgene presence. [Lane 1 corresponds to **(A,E)**, lane 2 to **(B,F)**, lane 3 to **(C,G)**, and lane 4 to **(D,H)]**. Self-regeneration correlated with presence of the *AtTCTP2-GFP* transgene (lanes 1–3); in a plant where no regeneration occurred *AtTCTP2* was not detected (lane 4). 18S rRNA was used as control for RNA integrity. Size bars = 1 cm.

Remarkably, **i**noculation of *A. rhizogenes* harboring *AtTCTP2* induced whole plant regeneration in tobacco (Figures 7A–E); this regeneration activity was quantitatively similar to *CmTCTP* (Hinojosa-Moya et al., 2013; Table 1). Interestingly, *AtTCTP1* was unable to induce regeneration to levels higher than background (Figure 7F; Table 1). In those cases in which there was plant regeneration, no *AtTCTP1-GFP* transgene was detected (not shown), while *GFP* and *35S* transgenes, and thus *AtTCTP2*, were amplified in all regenerated plants, indicating that regeneration requires the latter (Figure 7G). On the other hand, a construct in which the *AtTCTP2* start codon was replaced by a stop codon failed to induce regeneration above background levels, indicating that the protein is required for this phenomenon to occur (Table 1). Leaves from regenerated plants were also capable of regenerating plants themselves in the absence of exogenously applied plant hormones. Randomly selected leaves were excised from regenerated plants (four from each plant; five plants were analyzed) and placed on minimal MS medium. All these leaves gave rise to whole plants in three out of four cases; the exception corresponded to a plant that resulted negative for *GFP* and thus for *AtTCTP2* (Figure 8).

**Table 1:** **Percentage of regeneration induced by AtTCTP2 compared to AtTCTP1**.

**Construct**	**Rooting (%)**	**Plant regeneration (%) [plants regenerated/leaf explants]**	**Number of transgenic events per explant (±SD)**
Negative control	100	0 [0/350]	0
35S:*AtTCTP2* genomic *ORF-GFP*	100	40 [140/350]	4.5 ± 0.5
35S:*AtTCTP1-GFP*	85	0 [0/350]	0
35S:stop*AtTCTP2-GFP*	80	2 [2/100]	0
35S:stop*AtTCTP1-GFP*	90	1 [1/100]	0

**Figure 9:**
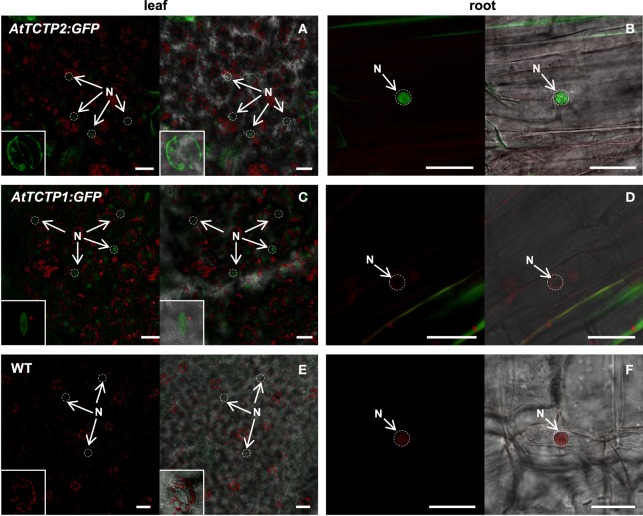
**Subcellular localization of AtTCTP2 and AtTCTP1 in regenerated tobacco plants**. Tissue from regenerated plants harboring both unmodified and modified versions of AtTCTP2 and AtTCTP1 were analyzed by confocal laser-scanning microscopy. **(A)** AtTCTP2-GFP accumulates in stomata (insert: bottom-left) and nuclei (N, arrowheads) in leaves; **(B)** while in roots its signal was associated to nuclei and vascular tissue (N, arrowheads). **(C)** AtTCTP1-GFP also accumulates in stomata (insert: bottom-left) and nuclei in leaves, but not in **(D)** root nuclei. **(E–F)** Representative wild-type controls for leaf and root are shown. Bars = 25 μm.

Both Arabidopsis TCTPs showed stomatal and nuclear localization in leaves. However, AtTCTP2 was readily detected in the nuclei of root cortical cells of regenerated plants, in contrast to AtTCTP1 (Figures 9A–D). Furthermore, *AtTCTP2:GFP* fusions driven by the CaMV35S or its endogenous promoter accumulated in punctae in the cell periphery, reminiscent of non-cell autonomous proteins, such as NtNCAPP1 (Lee et al., 2003; Figure S6). In contrast, in the few cases in which *AtTCTP1* could have induced regeneration, AtTCTP1-GFP fusions did not show such accumulation pattern, similar to the WT control (Figures 9C–F).

Another interesting feature observed in the progeny of regenerated plants was that they showed larger leaf area until 45 days after germination (Figure S7A), although by day 75 the size of these plants was comparable to non-transformed controls (Figure S7B). On the other hand, the percentage of regeneration was similar between different versions of the *AtTCTP2* construct (i.e., genomic ORF, fused to GFP, or without GFP; Figure S7C and Table S1).

### A sequence in AtTCTP1, absent in AtTCTP2, suppresses its regeneration capacity

**Figure 10:**
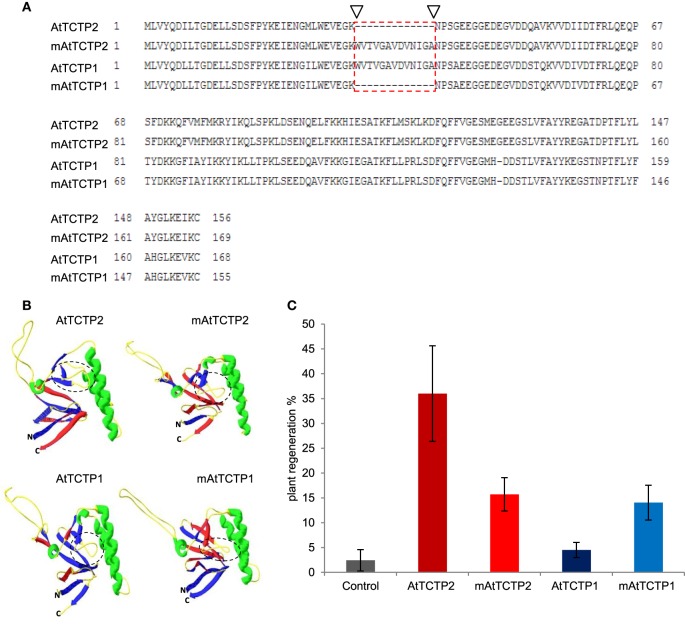
**Domain swapping between AtTCTP2 and AtTCTP1 alters their regeneration capacity. (A)** Amino acid sequence alignment of AtTCTP2 and AtTCTP1 versions with ClustalX. The main difference between unmodified (normal/endogenous) protein versions is located in the 13 amino acid region shown by arrowheads and red-dashed rectangle; this region was swapped between AtTCTP1 and AtTCTP2 in order to generate the modified versions for both proteins. **(B)** Predictive three-dimensional structures of AtTCTP2 and AtTCTP1 (unmodified and modified versions) were modeled with the SWISS-MODEL automated protein structure homology-modeling server and processed using Deep View program (Swiss-Pdb-Viewer); dashed circles underline the region with more structural differences in all four versions. **(C)** Rate of plant regeneration is altered when both AtTCTP2 and AtTCTP1 are modified. Transformation of tobacco explants was performed with the four-overexpression versions (fused to GFP) previously mentioned. Three transformation events were performed with 30 explants per biological replicate (*n* ≈ 90), given as mean ± SE. Plant regeneration rate of AtTCTP2 was reduced significantly while that of AtTCTP1 increased in the modified versions (mAtTCTP2 and mAtTCTP1) relative to the unmodified ones (AtTCTP2 and AtTCTP1).

**Figure 11:**
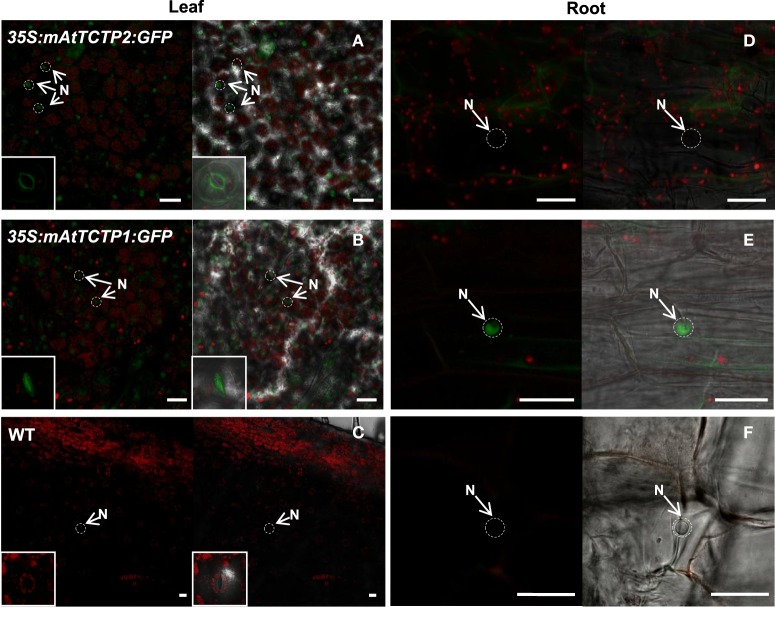
**Protein accumulation patterns are inverted in roots from regenerated plants due to domain swapping of AtTCTP2 and AtTCTP1**. Regenerated plants harboring both unmodified and modified versions of AtTCTP2 and AtTCTP1 fused to GFP were analyzed by confocal laser-scanning microscopy in leaves and roots. **(A,B)** In the case of the modified versions (mAtTCTP2 and mAtTCTP1), GFP signal was detected mostly in mesophyll nuclei (arrowheads) and stomata (insert: bottom-left); **(C)** background fluorescence in the WT control. **(D–F)** In roots the localization pattern was modified changed relative to the to the unmodified versions (AtTCTP2 and AtTCTP1, Figure 7), **(D)** mAtTCTP2 signal was no longer found in nuclei (N, arrowhead) in the midroot region. **(E)** In contrast, mAtTCTP1 nuclear accumulation was detected in the same tissue. **(F)** Wild-type controls in roots. Scale bars = 25 μm.

AtTCTP1 and AtTCTP2 share a ~80% similarity at the amino acid level, the most striking difference being a deletion of 13 amino acids in AtTCTP2 relative to AtTCTP1 (Figure 10A). Modified versions of these proteins were obtained in which this domain was deleted in AtTCTP1, and added in AtTCTP2. The predicted structure of mAtTCTP1 resembled AtTCTP2, while mAtTCTP2 resembled AtTCTP1 (Figure 10B). Regeneration assays in tobacco showed that, indeed, mAtTCTP1 behaved as AtTCTP2 and mAtTCTP2 as AtTCTP1 in terms of regeneration frequency (Figure 10C). Thus, while mATCTP1 regeneration activity increased from ~3 to ~13%, this decreased from ~35 to ~16% in mATCTP2 (Table S2). Furthermore, localization of these proteins was modified. Indeed, mAtTCTP1 was found in nuclei of roots of regenerated plants, while mAtTCTP2 localized to cytoplasm and, in a very small proportion, nuclei (Figure 11 and Figure S8). Thus, nuclear localization of AtTCTP1 and 2 in root cortical cells correlates with their capacity to induce plant regeneration. This was observed consistently in samples from independent transformants (Figure S8). It must be mentioned that the predicted structure of CmTCTP and AtTCTP2 are more similar between them than to AtTCTP1 (Figure S9).

Additional analyses indicate that *AtTCTP1* and *AtTCTP2* could have partially non-overlapping functions. Indeed, RT-qPCR assays under different growth conditions showed that while cytokinin, auxin, gibberellic acid, and brassinosteroid induce moderately accumulation of *AtTCTP1* mRNA, these suppress the accumulation of the *AtTCTP2* mRNA (Figure S10). Other metabolites and nutrients did not elicit a differential response.

Approximately ~40% of the progeny of *AtTCTP2*-regenerated plants harbored this gene (Table S3). This compares favorably with transformation efficiencies of plants using current methods. No regeneration was observed in tobacco when *AtTCTP2* was delivered via either *A. tumefaciens* or microprojectile bombardment (not shown).

## Discussion

Given the wide range of activities attributed to TCTP, and its pivotal role in regulation of growth and development, it is not surprising that null mutants show a lethal phenotype, as in the case of Drosophila, mouse, and Arabidopsis. It must be mentioned that the function of TCTP is known in only few organisms. Furthermore, in the case of several multicellular eukaryotes, their genomes harbor more than one *TCTP* gene, for which it is reasonable to assume that their function may not overlap completely. In Arabidopsis one of the *TCTP* genes, *AtTCTP1*, is expressed in most tissues at high levels. TCTP protein and/or mRNA have been found in the phloem translocation stream in several plant species, so it is possible that it functions in a non-cell autonomous manner. We have recently reported that the pumpkin *TCTP* (*CmTCTP*) mRNA and protein are also found in the phloem long-distance translocation stream; additionally, it promotes an important increase in biomass in cultured cells as well as cell size, and induces whole-plant regeneration in tobacco when expressed in *A. rhizogenes*. Our results indicate that *AtTCTP2* shares some of these features, in contrast to *AtTCTP1*. Complementation of an *AtTCTP2* knockout mutant supports the notion that this is a functional gene, essential for viability of Arabidopsis. The observed number of segregants from the complementation experiment deviated from the expected ratio. The CaMV 35S promoter drives the expression of the *AtTCTP2-GFP* construct used for complementation, but may not be able to do it efficiently during ovule development since it is expressed at low levels in this tissue. *AtTCTP2* is expressed in ovules and thus may be required for ovule development (see Figure 5). On a speculative note, it is possible that in homozygous *AtTCTP2* mutants complemented with *35S:AtTCTP2-GFP* the concentration of this protein is not sufficient for normal development to occur.

### *AtTCTP1* and *AtTCTP2* have different expression patterns and may function in different developmental stages

The fact that *AtTCTP2* mRNA was not detected before, and is only detectable after a more thorough denaturation suggests that it is rich in strong secondary structure, although the predicted ΔG of *AtTCTP2* mRNA is lower than that for *AtTCTP1* mRNA. This could explain the higher temperature and time required to denature it and thus synthesize the corresponding complementary DNA. Interestingly, the *AtTCTP2* gene lies in a double-stranded (ds) RNA hotspot, in contrast to *AtTCTP1* (Li et al., 2012), which raises the possibility that *AtTCTP2* mRNA contains secondary structure or occurs as dsRNA. Interestingly, in this work no reads were obtained for *AtTCTP2* mRNA; instead, small RNAs corresponding to this gene were found, supporting the notion that it is indeed expressed and may be regulated through gene silencing.

The *AtTCTP2* ORF sequence obtained in this study has been submitted to Genbank (accession number KR261461) and is identical to the one originally reported from the Arabidopsis genome project. Both sequences harbor a deletion of 13 amino acids relative to the *AtTCTP1* ORF. More recently this record has been replaced by yet another sequence that has the same length as *AtTCTP1*. Different *AtTCTP2* cDNA clones obtained in this study were sequenced; four were identical to the shorter version of this ORF, while another was identical to *AtTCTP1*; no sequence corresponding to the new curated version of *AtTCTP2* was obtained, suggesting that the shorter sequence is the biologically relevant. Nonetheless, it cannot be discarded that this mRNA is subject to alternative splicing giving rise to these two protein versions. Proteomic studies should help clarify this question.

It has been shown that *AtTCTP1* is expressed at much higher levels than *AtTCTP2* (~2–3 orders of magnitude) although it is probable that the latter was underestimated; after thorough denaturation, *AtTCTP2* RNA levels were found to be 60-fold higher than in untreated RNA from roots. Also, *AtTCTP2* expression is much more localized, in contrast to *AtTCTP1*, which is expressed almost constitutively (Berkowitz et al., 2008). The fact that *AtTCTP2* expression was observed in minor veins (but only in rosette leaves) suggests a role in vascular tissue function and may additionally be related to long-distance signaling. The observed segmented pattern of GUS activity in leaves is reminiscent to the pattern of other vascular-specific promoters in Arabidopsis, particularly in the case of the Arabidopsis *CmPP16* homolog, At3g55470, and At1g34260 (Ruiz-Medrano et al., 2011). Also, its expression in stomata could indicate a role in differentiation of this cell type, or, as in the case of *AtTCTP1*, response to water deficit (Kim et al., 2011). This gene could be also involved in ovule and trichome development, given the expression of the promoter in these cell types. Interestingly, while *AtTCTP2* was expressed in papillae from stigmata (and therefore could have a role in pollen recognition), its promoter is not active in anthers; in contrast, and as mentioned previously, *AtTCTP1* is required for male gametophyte development (Berkowitz et al., 2008; Brioudes et al., 2010). However, during seed development, no *AtTCTP2* promoter activity was detected in this tissue.

Importantly, the *AtTCTP2* promoter was active in secondary root primordia, but not in the apex, suggesting a more direct role in differentiation than in proliferation, at least in this tissue. This pattern is markedly different from the *AtTCTP1* promoter, which at early stages of development is inactive in the primary root, except for the elongation zone and meristem in both primary and lateral roots, which is suggestive of a role in proliferation (Kim et al., 2011). In contrast, the specific pattern observed for the *AtTCTP2* promoter suggests that this gene is involved in creating the boundary in root cortex that results in secondary roots; also, no signal was observed in root meristems. Of note, the expression pattern found for this gene promoter is strikingly similar to that of *PUCHI*, which is a transcription factor involved in the early stages of lateral root morphogenesis in Arabidopsis (Hirota et al., 2007), as well as in regulation of floral meristem identity (Karim et al., 2009). Thus, a similar role for *AtTCTP2* is possible.

Heterozygous plants for the SALK_045146 T-DNA insertion show intermediate phenotypes relative to homozygous mutants. This correlates with *AtTCTP2*, but not *AtTCTP1*, mRNA levels. While the underlying mechanism is not clear, it is possible that an aberrant fragment of the *AtTCTP2* transcript could be synthesized, which could in turn eventually trigger PTGS of the WT allele. Variable *AtTCTP2* transcript levels in different heterozygous lines may result from epigenetic mechanisms, i.e., silencing of the WT allele, although at varying levels between independent lines.

The accumulation pattern of *AtTCTP1* and *AtTCTP2* transcripts differ in response to different treatments. While the *AtTCTP1* mRNA is moderately induced by several of these treatments, in most cases the *AtTCTP2* mRNA levels decrease, supporting the notion that their functions are not completely equivalent.

### *AtTCTP1* and *AtTCTP2* functions show little overlap

Our results indicate that *AtTCTP2* is essential for plant viability, and that *AtTCTP1* and *AtTCTP2* function in Arabidopsis may not be redundant. Indeed, *AtTCTP1* null mutants are lethal in early embryonic development (Berkowitz et al., 2008; Brioudes et al., 2010), while seeds from *AtTCTP2* T-DNA insertion mutants are able to germinate and reach the early rosette stage, after which they rapidly undergo necrosis and die. Thus, *AtTCTP1* regulates earlier stages of development than *AtTCTP2*; if the latter is nonfunctional, the other gene conceivably cannot compensate for its loss. This suggests that *AtTCTP2* functions in later stages of development. We have recently shown that *AtTCTP2* mRNA and protein move long-distance through a graft union in tobacco (Toscano-Morales et al., 2014); *AtTCTP1* mRNA is also highly mobile, as has been demonstrated between Cuscuta and Arabidopsis and within Arabidopsis (Kim et al., 2014; Thieme et al., 2015). Expression in stomata may be related to response to water deficit, as in the case of *AtTCTP1*. An important result is that heterozygous plants showed an intermediate phenotype. Silencing of the *AtTCTP1* gene, presumably because an aberrant *AtTCTP2* mRNA could still be generated, which could trigger the degradation of this and of *AtTCTP1* mRNA, is unlikely to occur since this transcript accumulates to levels even higher than wild type controls.

*AtTCTP2* was able to enhance regeneration of tobacco plants when harbored by *A. rhizogenes*, in contrast to *AtTCTP1*. We observed that such regeneration takes place through direct formation of plantlets from the explants. Although more work is required, it appears that somatic embryogenesis does not occur in this case. It must be mentioned that regeneration does not occur naturally in Arabidopsis. However, lateral root development and plant regeneration are related, so it is possible that *AtTCTP2* has a role in this process, but in heterologous systems (such as tobacco) it can induce regeneration; indeed, induction of regeneration in Arabidopsis occurs through a root developing pathway (Sugimoto et al., 2010). Indeed, Arabidopsis in which *AtTCTP2* was silenced developed less lateral roots; however, this effect is more pronounced in plants in which *AtTCTP1* was silenced, suggesting a role in lateral root formation (Figure S4). Proteins that are structurally related to AtTCTP2, in the appropriate environment, could also induce regeneration (see below). Since *AtTCTP2*-mediated regeneration requires *A. rhizogenes*, it can be assumed that genes encoded by the Ri plasmid are likely required for plant regeneration from roots. It is possible that auxins are involved in plant regeneration since this plasmid harbors genes involved in their synthesis. Likewise, it is also possible that *rol* genes are necessary to create an environment favorable for regeneration. Since regeneration correlates with expression of *AtTCTP2* (and *CmTCTP*) in roots, it is possible that some plant TCTP isoforms may be involved in vegetative reproduction in those species in which this occurs through root and stem structures, such as stolons and tubers.

No extant plant TCTPs harbor the deletion observed in AtTCTP2 relative to AtTCTP1, with the exception of *Arabidopsis lyrata*. However, the fact that the phloem-transported CmTCTP shows the same regeneration capacity as AtTCTP2 indicates that other isoforms may have similar functions (Hinojosa-Moya et al., 2013). It must be mentioned that the deletion of AtTCTP2 relative to other TCTP isoforms falls within the flexible loop, which shows the highest variability between members of this family. The *Giardia lamblia* TCTP displays a similar deletion relative to other TCTP sequences. The results obtained in the present work also indicate that AtTCTP2-mediated regeneration occurs only via *A. rhizogenes*, which requires the localization of this protein in nuclei of root cortex. The targeting of AtTCTP1 to root nuclei was sufficient to increase regeneration efficiency by this protein.

The predicted structure of CmTCTP and AtTCTP2 resemble each other and, interestingly more than AtTCTP1 (Figure S9). This suggests that the capacity for plant regeneration, and thus vegetative reproduction, of some TCTP isoforms may have arisen through independent mechanisms, via substitution of residues in the extra domain of AtTCTP1 relative to AtTCTP2, or through deletion of this sequence, as in the case of *AtTCTP2* and its homolog in *A. lyrata*. Plants harbor variable numbers of *TCTP* genes. Certain species harbor only one *TCTP* gene, suggesting that in some instances it performs the function of both TCTP proteins in Arabidopsis. Evidently, such regeneration capacity has to be confirmed experimentally in other plant species, but the assay presented in this work can be readily used to determine whether different TCTP genes from a given plant species are involved in regulation of differentiation. A predictive analysis of plant TCTPs structure suggest that there may be indeed two types of TCTPs; some harbor an AtTCTP1-like structure, while others a CmTCTP-like (and thus an AtTCTP2-like) structure (Gutiérrez-Galeano et al., 2014). Interestingly, a similar distinction could be made in other eukaryotes regarding TCTP structure. Indeed, TCTPs from human and *Schizosaccharomyces pombe* show similarity to AtTCTP1, while the *Plasmodium falciparum* TCTP (PfTCTP), as well as possibly from other blood-borne parasites, shows a structure resembling that of CmTCTP and AtTCTP2 (Gutiérrez-Galeano et al., 2014). Furthermore, PfTCTP is incorporated more efficiently into B cells than the human TCTP, but induces a much lower proliferation rate than the latter, suggesting a role for this protein in the observed suppression of memory B cells in malaria (Calderón-Pérez et al., 2014). This supports the notion that the aforementioned structural differences between TCTPs may have a functional role, albeit diverse.

TCTP is found in most eukaryotes; interestingly, the only exceptions found are certain green algae. Indeed, a BLAST search for TCTP homologs failed to identify them in chlorophytes whose genomes have been sequenced, except for *Coccomyxa subellipsoidea* (not shown). The absence of this gene in these species, if confirmed, could have interesting implications regarding the evolution of land plants.

TCTP in mammals is involved in cell reprogramming. Indeed, breast cancer cell revertants show a dramatic decrease in TCTP expression, and silencing of TCTP expression in mouse mammary epithelial tumor cells also leads to a revertant phenotype (Tuynder et al., 2002; Amson et al., 2012). Reprogramming is likely required for plant regeneration from non-differentiated tissue or tissue explants. Our results suggest that AtTCTP2 may have an important role in such process.

## Concluding remarks

Members of the TCTP superfamily play vital roles regarding the control of general growth and development. It has been found to show multiple functions at the molecular level in model animal organisms, but its role is less clear in plants. In this work we found that the Arabidopsis *AtTCTP2* is not a pseudogene, that is essential for viability, and that it enhances plant regeneration. The latter could reflect a role for this gene in differentiation. Finally, *AtTCTP1* cannot compensate for the inactivation of the *AtTCTP2* gene and vice versa, suggesting functional non-redundancy.

## Author contributions

RT performed most of the experiments; RR-M, RT, and BX-C devised the experimental plan, wrote the manuscript and obtained financial support for the work; RGG-G helped in designing the domain-swapping experiments, and JJH-M and JLC-P helped with the NT1 cell proliferation assay and supervised the tobacco regeneration experiments. JLR-S carried out the dd-PCR assay, and SVG-G carried out the qRT-PCR after different treatments. All authors read and approved the final version of the manuscript.

### Conflict of interest statement

The authors declare that the research was conducted in the absence of any commercial or financial relationships that could be construed as a potential conflict of interest.

## Supplementary material

Figure S1 **(A)** Analysis of T-DNA insertion copy number by ddPCR in two SALK_045146 heterozygous lines and a WT control. **(B)** Average of photosynthetic rate from several (+/−) lines compared to WT. The global average of CO_2_ assimilation of all (+/−) plants shows no significant difference compared to WT plants. Eleven biological replicates were performed, given as means ± SE.

Figure S2 ***AtTCTP2* mRNA may have a high degree of secondary structure**. Total RNA of the different samples (leaves and roots) was thermally treated at 75°C for 10 min followed by qRT-PCR for both *AtTCTP2* and *AtTCTP1* transcripts and compared with the same untreated samples. *AtTCTP2* transcript levels increased 28-fold in leaves and 64 fold in roots with the treatment, compared to a three-fold increase for *AtTCTP1* in both tissue samples. Three biological replicates were performed, given as means ± SE.

Figure S3 ***AtTCTP2* mRNA accumulates at higher levels in stems and cauline leaves**. qRT-PCR for each tissue was relative to leaf expression and first normalized against reference genes (18S rRNA and actin), three biological replicates were performed, given as means ± SE. **(A)**, qRT-PCR results for *AtTCTP2* mRNA; **(B)**, qRT-PCR results for *AtTCTP1* mRNA.

Figure S4 **Effect of silencing of *AtTCTP1* and *AtTCTP2* genes on Arabidopsis development. (A)** Phenotype of WT controls vs. *AtTCTP2*-RNAi and **(B)** *AtTCTP1*-RNAi lines, respectively, 15 dag on MS medium. **(C)** *AtTCTP2* and *AtTCTP1* transcript levels for each RNAi line tested (with 18S RNA as endogenous control) by qRT-PCR using the ΔΔCT method compared to WT controls. **(D)** Germination rate, **(E)** leaf area, and **(F)** root length of *AtTCTP2*-RNAi, *AtTCTP1*-RNAi, and WT lines; 30 plants per line were analyzed 15 dag.

Figure S5 ***AtTCTP2* expression in calli derived from NT1 cells increases biomass accumulation**. Dry weight kinetics of tobacco calli was followed for 21 days after bombardment with control (empty vector) and *AtTCTP2*-overexpressing construct. Differences in dry weight were measured at days 1, 7, 14, and 21 after calli transformation. Kinetics of transformed calli was followed during 21 days (*n* = 20 total) relative to wild type (*n* = 20); six biological replicates were performed, given as means ± SE.

Figure S6 **Confocal laser scanning microscopy image of a mesophyll cell expressing a *AtTCTP2-GFP* fusion driven by the *CaMV 35S* promoter (left) and the *AtTCTP2* promoter (right)**. Green fluorescence was observed in endosomes (arrowheads) but also in the cell periphery. Size bars = 50 μM.

Figure S7 **Phenotypic analysis of Progeny (F1) from each overexpressing (OX) line. (A)** Plants analyzed 45 days after germination showed a higher size compared to wild-type. **(B)** However, plants with 75 days after germination, the size of all the transgenic lines were comparable to the WT control. Bars = 5 cm. **(C)** Plant regeneration assay was performed for each *AtTCTP2* transgenic line. Three independent transformation events were carried out for a total of 100 explants per construct (*n* = 100), given as mean ± SE. Regeneration percentage was measured 28 days after transformation.

Figure S8 **Percentage of GFP positive nuclei detected in the midroot region of transgenic tobacco plants harboring endogenous and modified overexpression versions of *AtTCTP2-GFP* and *AtTCTP1-GFP***. Confocal microscopy was used to determine the number of GFP positive nuclei in the midroot region of samples. The visualization and nuclei count was performed in three different visual sections in each root, examining three roots per sample in a total of three different samples per construct, giving a total of *n* = 27 scanning series analyzed for each case, given as mean ± SE.

Figure S9 **Predictive three-dimensional structures of AtTCTP2, CmTCTP, and AtTCTP1**. Modeling was made with SWISS-MODEL (see experimental procedures). Dashed circles indicate regions with more structural similarities between AtTCTP2 and CmTCTP, compared to AtTCTP1.

Figure S10 **Effect of different treatments on *AtTCTP1* and *AtTCTP2* transcript levels determined by qRT PCR. (A)** mRNA levels of both *TCTP* genes from Arabidopsis in response to exogenous hormones [kinetin (cytokinin), IAA (auxin), GA (gibberellins), ABA (abcisic acid), BRAS (brassinosteroids)]. **(B)** mRNA levels of *AtTCTP1* and *AtTCTP2* in response to several exogenous substrates [mannitol (osmotic stress), PEG (drought)], salts [NaCl, KCl, KNO_3_ (salinity stress)], mineral nutrient (phosphate concentrations-NaH_2_PO_4_), and 4°C (cold stress). In most cases, two different concentrations were tested, and three independent biological replicates were performed per sample (three plants pooled); each sample was subjected to three technical replicates given as mean ± SE. Concentrations tested are shown in each case at the bottom of the graphs.
