# Supplementary material for: AtTCTP2, an Arabidopsis thaliana homolog of Translationally Controlled Tumor Protein, enhances in vitro plant regeneration
Source: Front Plant Sci. 2015 Jul 2;6:468. doi: 10.3389/fpls.2015.00468 (PMC4489097; doi:10.3389/fpls.2015.00468)

# Figure S1
Toscano-Morales *et al.*, 2015

**A**

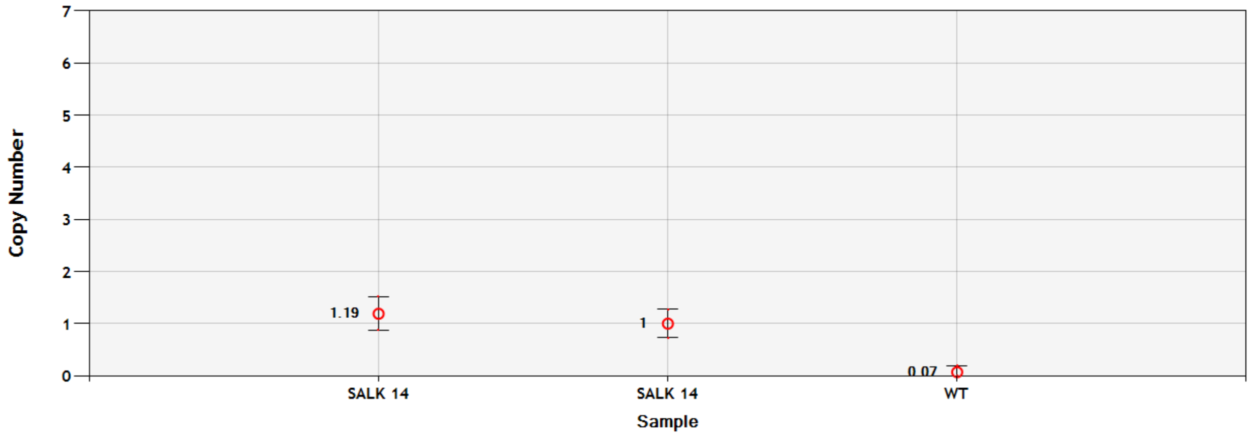


**B**

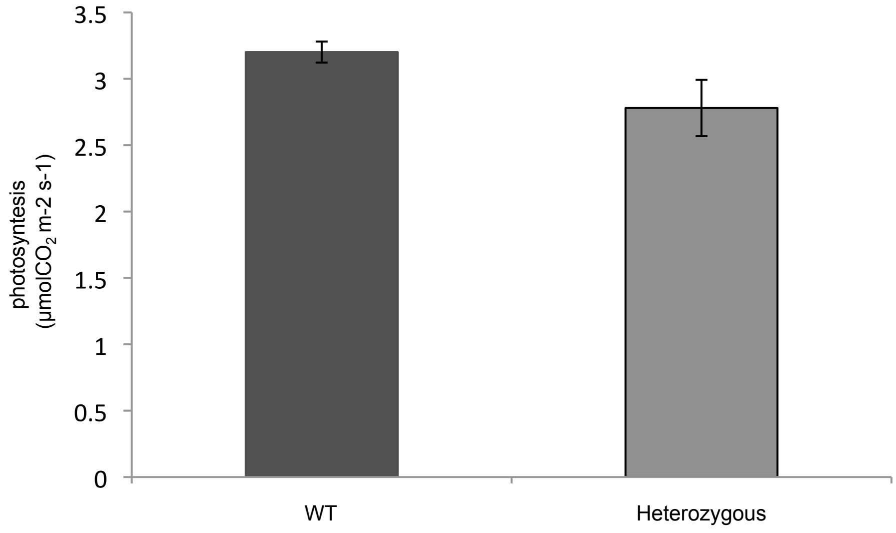

# Figure S2

Toscano-Morales *et al*., 2015

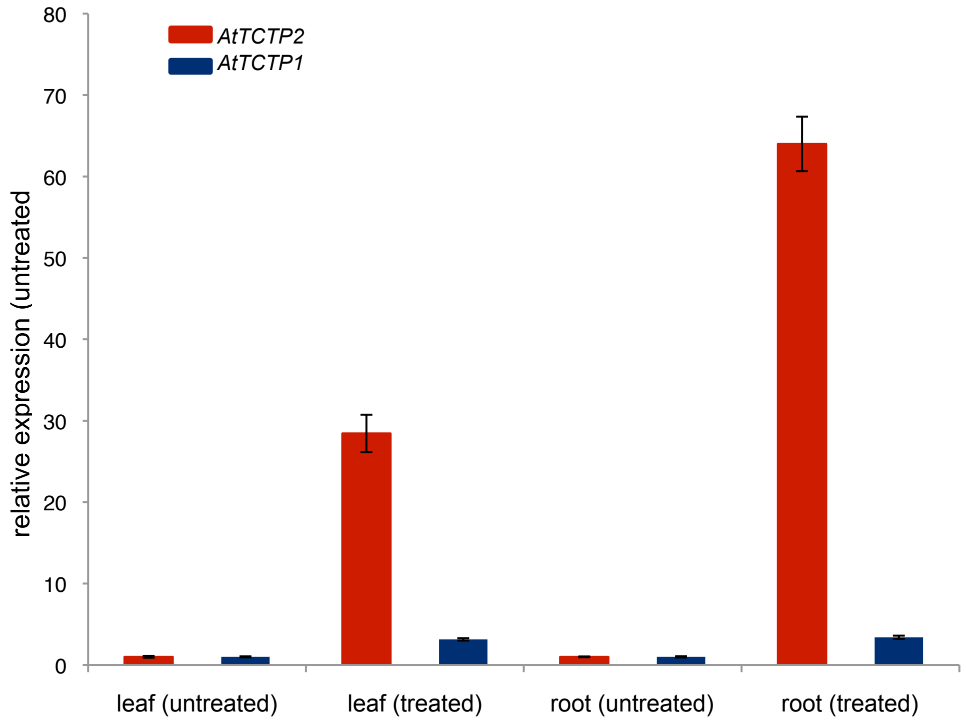

Figure S3

Toscano-Morales *et al.*, 2015

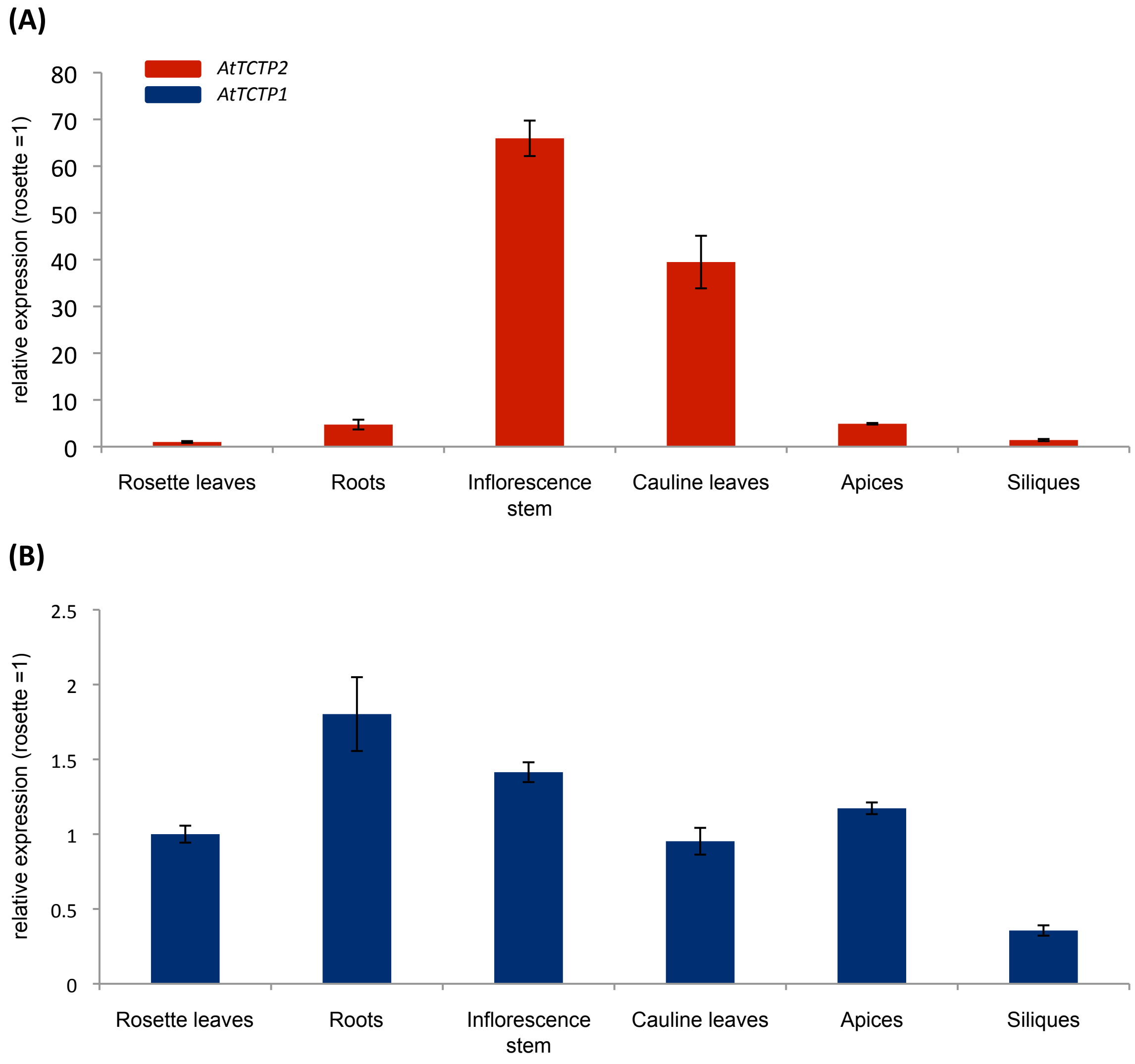

# Figure S4
Toscano-Morales *et al.*, 2015

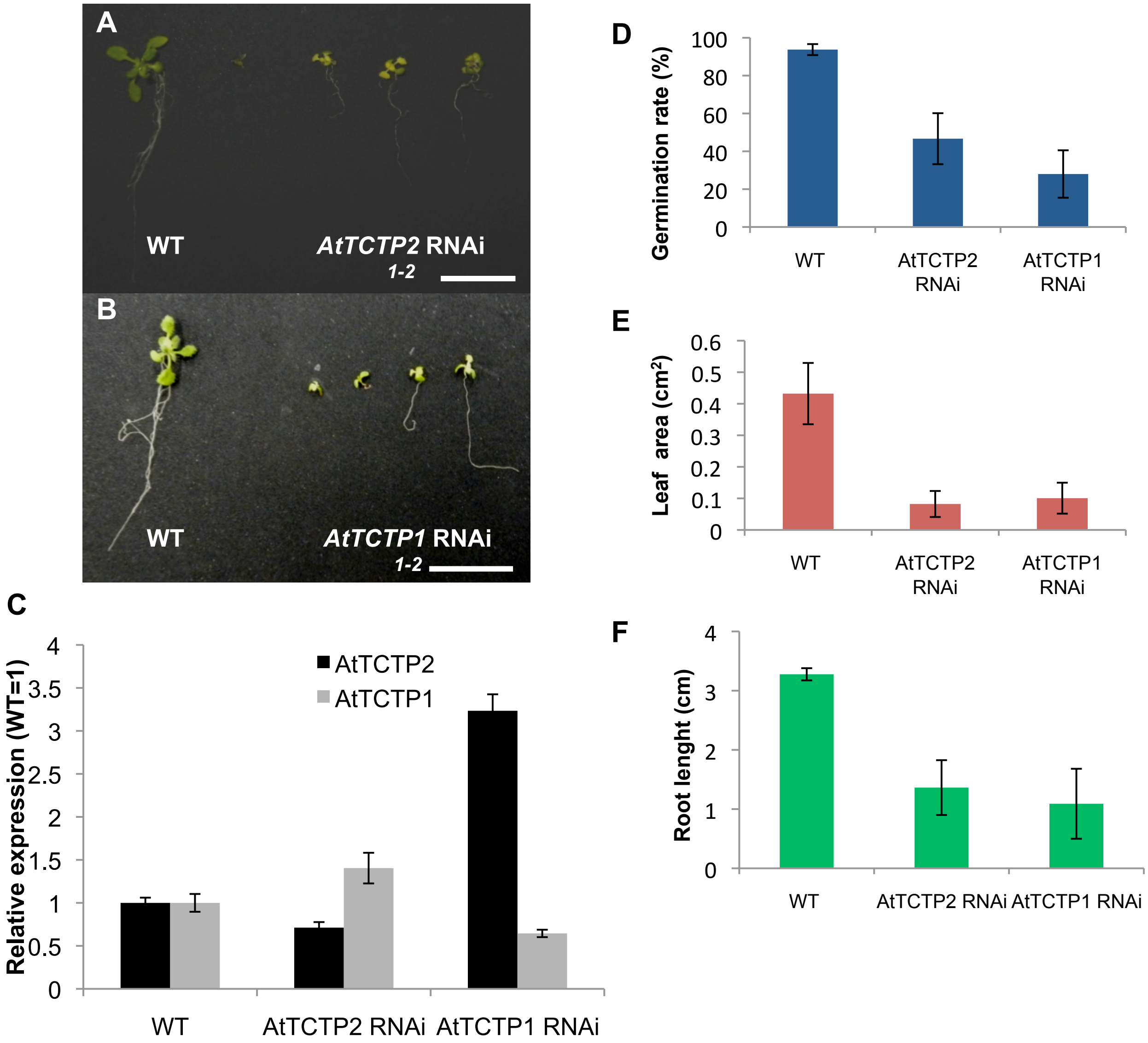

# Figure S5
# Toscano-Morales *et al*., 2015

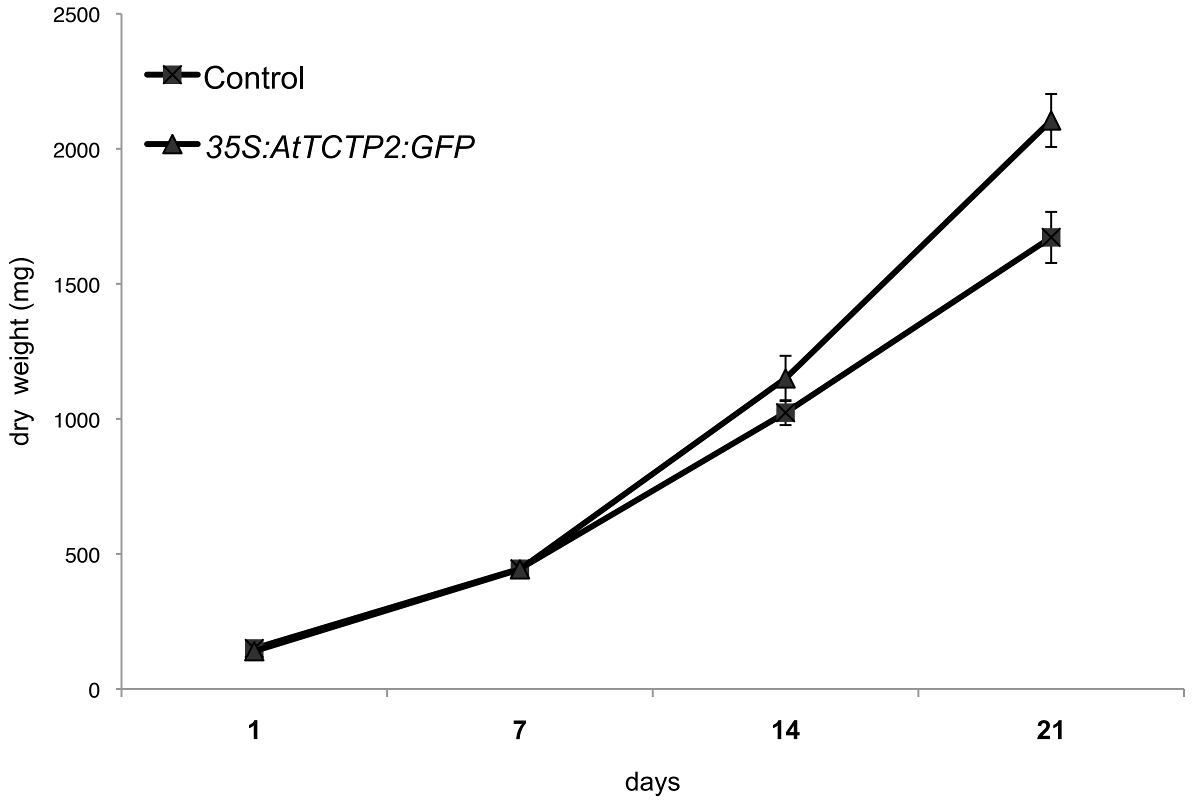

Figure S6

Toscano-Morales *et al*., 2015

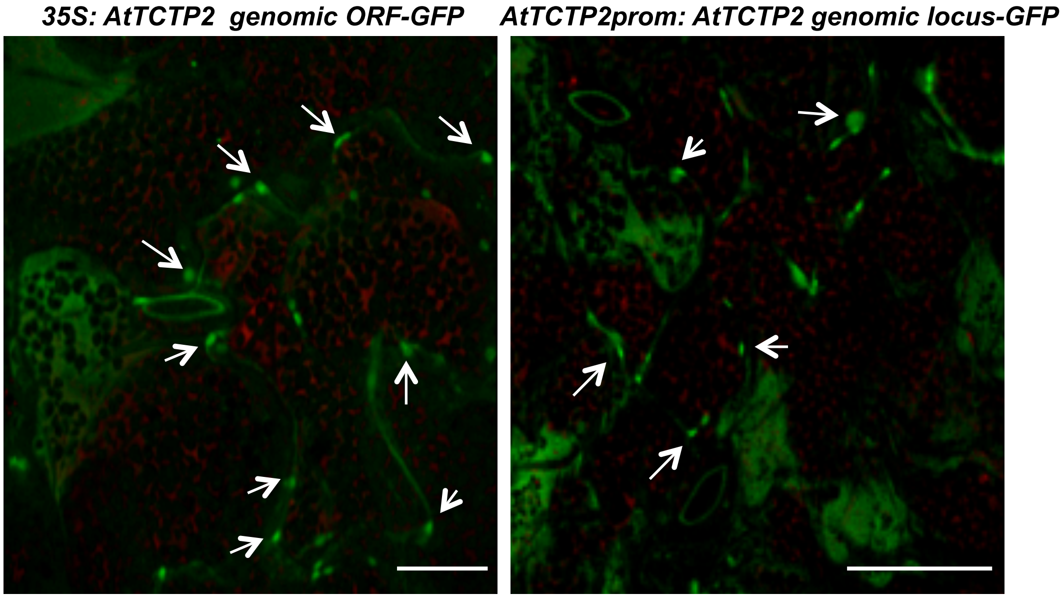

Figure S7
Toscano-Morales *et al.*, 2015

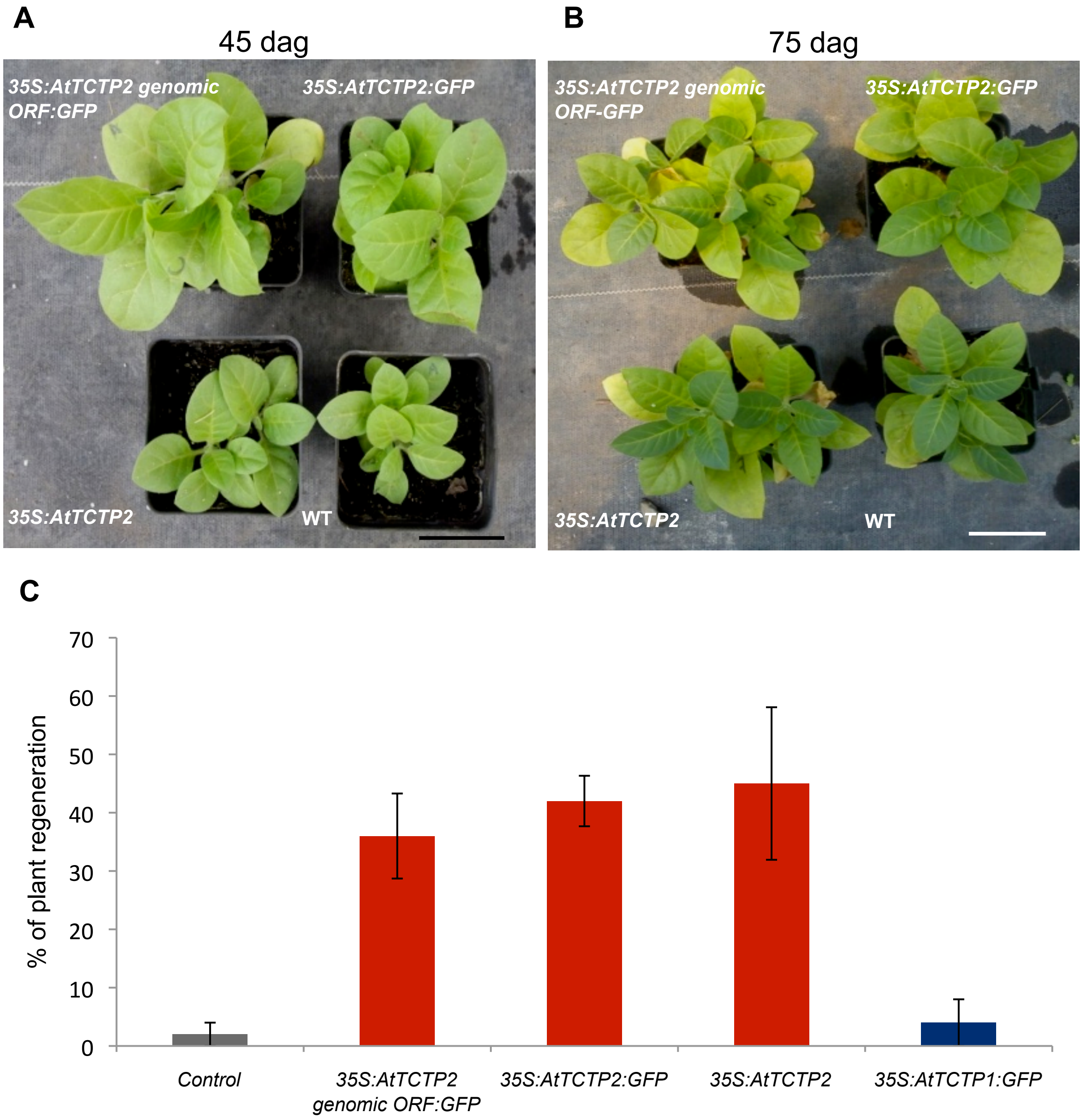

# Figure S8
Toscano-Morales *et al*., 2015

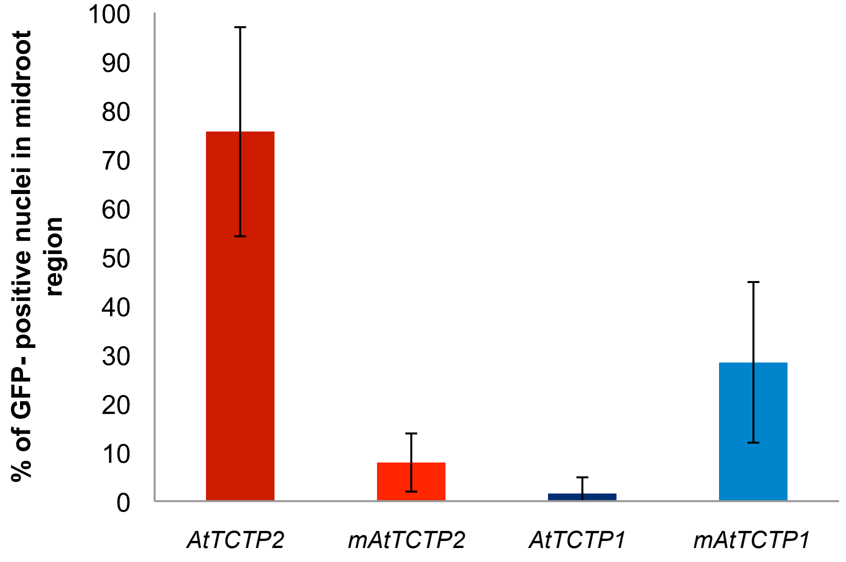

# Figure S9
Toscano-Morales *et al*., 2015

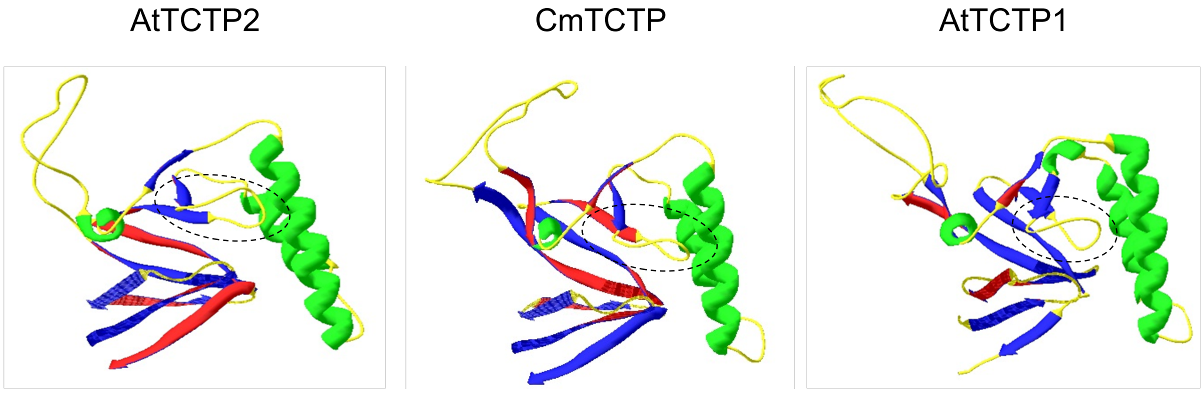

# Figure S10
Toscano-Morales *et al.*, 2015

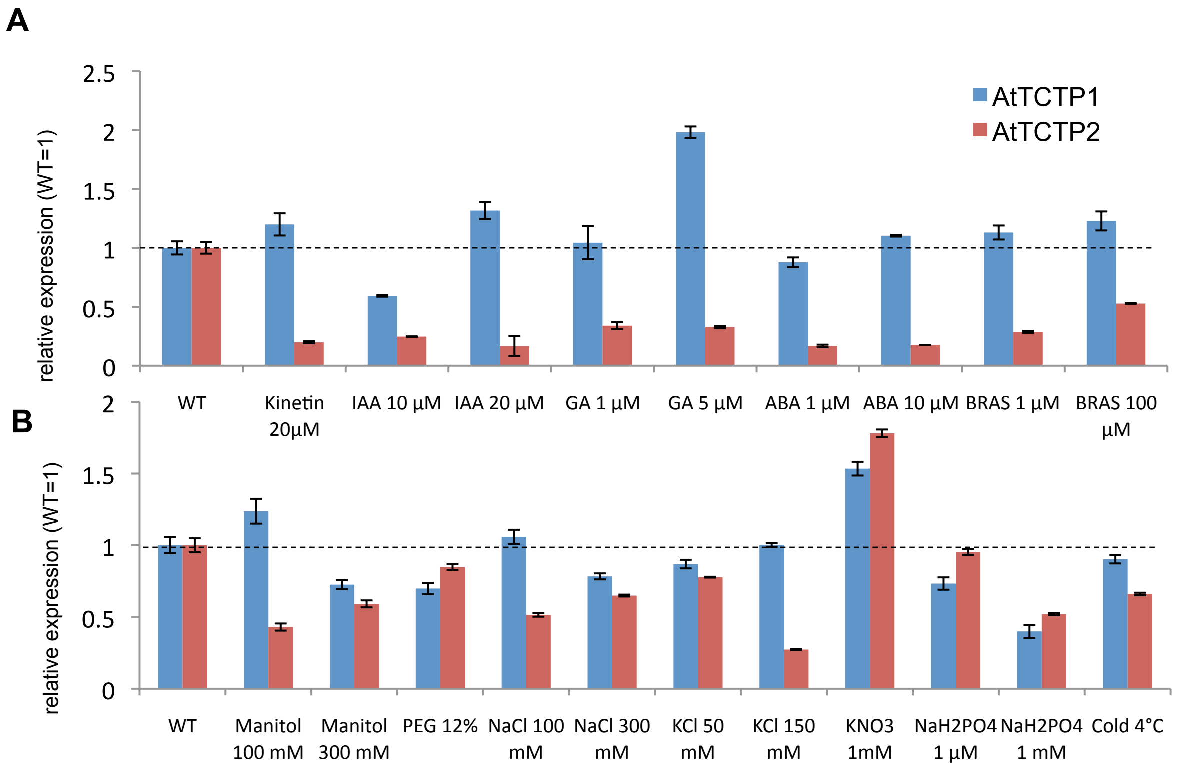

Supplement: Figure S1 — (A) Analysis of T-DNA insertion copy number by ddPCR in two SALK_045146 heterozygous lines and a WT control. (B) Average of photosynthetic rate from several (+/−) lines compared to WT. The global average of CO2 assimilation of all (+/−) plants shows no significant difference compared to WT plants. Eleven biological replicates were performed, given as means ± SE. [file Image1.PDF]
