# Supplementary material for: AtTCTP2, an Arabidopsis thaliana homolog of Translationally Controlled Tumor Protein, enhances in vitro plant regeneration
Source: Front Plant Sci. 2015 Jul 2;6:468. doi: 10.3389/fpls.2015.00468 (PMC4489097; doi:10.3389/fpls.2015.00468)
Supplement: Supplementary file 2 [file DataSheet1.DOCX]

**Supporting tables**

**Table S1.** Percentage of regeneration induced by tobacco transformation with three different overexpression constructs

| Construct | % of plant regeneration  [regenerated plants^+^SD / leaf explants] |
| --- | --- |
| *Negative control* | 2% [2^+^ 2/100]* |
| *35S::AtTCTP2 genomic locus-GFP* | 36%[36^+^10/100] |
| *35S::AtTCTP2-GFP* | 42%[42^+^5/100] |
| *35S::AtTCTP2* | 45% [45^+^17/100] |
| *35S::AtTCTP1-GFP* | 4%^+^ [4^+^4/100]* |

**Table S2.** Percentage of regeneration induced by modified and unmodified versions of AtTCTP2 and AtTCTP1

| Construct | % of plant regeneration  [regenerated plants ^+^SD / leaf explants] |
| --- | --- |
| *Negative control (K599)* | 2.3% [2^+^2/85] |
| *35S: AtTCTP2-GFP* | 36.4% [31^+^8/85] |
| *35S:mAtTCTP2-GFP* | 15.8% [13^+^3/82] |
| *35S:AtTCTP1-GFP* | 4.6% [4^+^1.5/87] |
| *35S:m AtTCTP1-GFP* | 13.95% [12^+^3/86] |

**Table S3.** Percentage of transgenic seed (F1) harbored from regenerated plants

| Construct | % Regenerated plants (regenerated/total) | % transgenic plants regenerated (transgenic/total regenerated) | % F1 transgenic plants (GFP positive/total) |
| --- | --- | --- | --- |
| *35S:AtTCTP2*  *genomic ORF:GFP* | 40% (20/50) | 100% (20/20) | 40% (10/25) |
| *35S:AtTCTP2 ORF:GFP* | 42% (21/50) | 95% (19/21) | 44% (11/25) |
| *35S:AtTCTP1 ORF:GFP* | 4% (2/50) | 0% [0/2] | --- |

**Table S4**. List of primers used in this study.

| Primers | Gene | Primer Sequence (5’-3’) | Experiment/Assay |
| --- | --- | --- | --- |
| AtTCTP2 F | AtTCTP2 | ATGTTGGTCTACCAGGATATTCTTACA | Gene cloning |
| AtTCTP2-non-stop R | AtTCTP2 | GCACTTGATCTCTTTCAAGCCGTAGGC | Gene cloning |
| AtTCTP2-stop R | AtTCTP2 | TCAGCACTTGATCTCTTTCAAGCCGTA | Gene cloning |
| AtTCTP2-utr5’ F | AtTCTP2 | ATTTAGGTGAATGGAACATGGTTTTCA | Gene cloning |
| AtTCTP2-utr3’ R | AtTCTP2 | CGCCTGATCTCTCTCTCTCATCATTTA | Gene cloning |
| AtTCTP1 F | AtTCTP1 | ATGTTGGTGTACCAAGATCTTCTCACC | Gene cloning |
| AtTCTP1-non-stop R | AtTCTP1 | GCACTTGACCTCCTTCAAACCATGAGC | Gene cloning |
| AtTCTP2 MUT STOP | AtTCTP2 | TAGATGTTGGTCTACCAGGATATTCTT | Gene cloning |
| LP | AtTCTP2 | GCCTTTTTCGTTGCACTAATC | Genotyping |
| RP | AtTCTP2 | GATGATCCTTTGGGGAGTTTG | Genotyping |
| LBb1 | T-DNA | GCGTGGACCGCTTGCTGCAACT | Genotyping |
| *AtTCTP2* Sil F | AtTCTP2 | GGAGTTTTACTTTTTGTTAGTACTTTTGG | PTGS |
| *AtTCTP2* Sil R | AtTCTP2 | CAGAAATTTTTAAGAGAATTCACTTATCAATCTC | PTGS |
| *AtTCTP1* Sil F | AtTCTP1 | GAGAGAAGCTCTCGTTGGGTTACTGTG | PTGS |
| *AtTCTP1* Sil R | AtTCTP1 | CCAAAATTCAACTAATTGTAAATGATAAACCG | PTGS |
| P-AtTCTP2 F | AtTCTP2 | AAACCATCACCTTCGTCGCCATCGGCA | Promoter region |
| P-AtTCTP2 R | AtTCTP2 | GTTTGAAGAGAGTTAAAAGAGACTTTT | Promoter region |
| AtTCTP2-q F | AtTCTP2 | CTTTTCTCTACTTGGCCTACGGCTTGAA | Quantitative RT-PCR |
| AtTCTP2-q R | AtTCTP2 | AGGAAATTGCATAAAAGTTAACTTCACT | Quantitative RT-PCR |
| AtTCTP1-q F | AtTCTP1 | CATTTTTGTACTTCGCTCATGGTTTGAA | Quantitative RT-PCR |
| AtTCTP1-q R | AtTCTP1 | CGACGAAACACAGGACAATATAAAGAAACATA | Quantitative RT-PCR |
| 18 S-q F | 18S | GCCCGGGTAATCTTTGAAATTTCAT | Quantitative RT-PCR |
| 18 S-q R | 18S | GTGTGTACAAAGGGCAGGGACGTA | Quantitative RT-PCR |
| Actin-q F | Actin 8 | CCATGACGGGATCACATTTC | Quantitative RT-PCR |
| Actin-q R | Actin 8 | CAAACGCTGTAACCGGAAAG | Quantitative RT-PCR |
| GFP F | GFP | ATGGTGAGCAAGGGCGAGGAGCTG | Transgene detection |
| GFP R | GFP | CCTTGTACAGCTCGTCCATGC | Transgene detection |
| 35S F | 35S | GACTAGAGCCAAGCTGATCTCCTTTGCC | Transgene detection |
| 35S R | 35S | TCGACTAGAATAGTAAATTGTAATGTTG | Transgene detection |
| 35S ddPCR F | 35S | GAGACTTTTCAACAAAGGG | ddPCR-copy number |
| 35S ddPCR R | 35S | TCCACTATCTTCACAATAAAG | ddPCR-copy number |
| 35S ddPCR Probe | 35S | [FAM]-CCTCCTCGGATTCCATTGCC-[BHQ1] | ddPCR-copy number |
| HMGAt ddPCR F | HMGB1 | CAGAAAGGTGGGAAAGAGGA | ddPCR-copy number |
| HMGAt ddPCR R | HMGB1 | TTTGGTTTGTTTGGGTCCTT | ddPCR-copy number |
| HMGAt ddPCR Probe | HMGB1 | [HEX]-AGGCTTCTCAGCCGGTGCCT-[BHQ1] | ddPCR-copy number |
